# Plasmon‐Enhanced Single Extracellular Vesicle Analysis for Cholangiocarcinoma Diagnosis

**DOI:** 10.1002/advs.202205148

**Published:** 2023-01-25

**Authors:** Mi Ho Jeong, Taehwang Son, Yoo Keung Tae, Chan Hee Park, Hee Seung Lee, Moon Jae Chung, Jeong Youp Park, Cesar M. Castro, Ralph Weissleder, Jung Hyun Jo, Seungmin Bang, Hyungsoon Im

**Affiliations:** ^1^ Center for Systems Biology Massachusetts General Hospital Boston MA 02114 USA; ^2^ Division of Gastroenterology Department of Internal Medicine Severance Hospital Yonsei University College of Medicine Seoul 03722 Republic of Korea; ^3^ Cancer Center, Massachusetts General Hospital Harvard Medical School Boston MA 02114 USA; ^4^ Department of Radiology Massachusetts General Hospital Boston MA 02114 USA; ^5^ Department of Systems Biology Harvard Medical School Boston MA 02115 USA

**Keywords:** biomarkers, cholangiocarcinoma, diagnostics, extracellular vesicles, plasmonics

## Abstract

Cholangiocarcinoma (CCA) is a fatal disease often detected late in unresectable stages. Currently, there are no effective diagnostic methods or biomarkers to detect CCA early with high confidence. Analysis of tumor‐derived extracellular vesicles (tEVs) harvested from liquid biopsies can provide a new opportunity to achieve this goal. Here, an advanced nanoplasmonic sensing technology is reported, termed FLEX (fluorescence‐amplified extracellular vesicle sensing technology), for sensitive and robust single EV analysis. In the FLEX assay, EVs are captured on a plasmonic gold nanowell surface and immunolabeled for cancer‐associated biomarkers to identify tEVs. The underlying plasmonic gold nanowell structures then amplify EVs’ fluorescence signals, an effective amplification process at the single EV level. The FLEX EV analysis revealed a wide heterogeneity of tEVs and their marker levels. FLEX also detected small tEVs not detected by conventional EV fluorescence imaging due to weak signals. Tumor markers (MUC1, EGFR, and EPCAM) are identified in CCA, and this marker combination is applied to detect tEVs in clinical bile samples. The FLEX assay detected CCA with an area under the curve of 0.93, significantly better than current clinical markers. The sensitive and accurate nanoplasmonic EV sensing technology can aid in early CCA diagnosis.

## Graphical abstract

An advanced nanoplasmonic sensing technology is developed for the molecular analysis of single extracellular vesicles (EVs). Periodic gold nanowell arrays, fabricated by wafer‐scale interference lithography, significantly enhance fluorescence signals of tumor‐derived EVs, enabling sensitive detection of tumor‐derived EVs in human bile samples for cholangiocarcinoma diagnosis.

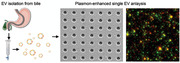

## 1 Introduction

Cholangiocarcinoma (CCA) is a fatal malignancy with a 5‐year survival rate below 20%.^[^ ^1^ ^]^ Along with pancreatic cancer, they are the only two cancers with increasing incidence and mortality rates. The high mortality rate is attributed to CCA's aggressiveness, late diagnosis, and refractoriness to chemotherapy.^[^ ^2^ ^]^ When present, CCA most commonly manifests as biliary obstruction, but clinical hurdles remain for diagnosis with endoscopic retrograde cholangiopancreatography (ERCP) or percutaneous biliary drainages. With ERCP, it has been reported that up to 20% of brush biopsies are found inconclusive, requiring repeated procedures and delaying treatment.^[^ ^3^, ^4^ ^]^ The only diagnostic biomarker currently recommended for CCA is carbohydrate antigen 19‐9 (CA19‐9), but it also has suboptimal sensitivity and specificity, ranging 70–80%.^[^ ^5^, ^6^ ^]^ The CA19‐9 level can be elevated even with benign biliary structures, especially for patients with obstructive jaundice. Therefore, CA19‐9 alone is insufficient to distinguish between malignant and benign cases.^[^ ^6^, ^7^ ^]^

Bile contains secretomes from CCAs, such as proteins,^[^ ^8^, ^9^ ^]^ nucleic acids,^[^ ^10^, ^11^ ^]^ cytokines,^[^ ^12^ ^]^ and extracellular vesicles (EVs).^[^ ^13^, ^14^, ^15^ ^]^ More sensitive ways of bile analysis could provide unique opportunities to screen and identify new biomarkers, as the biofluid likely contains biomarkers shed from CCA cancer cells locally. Recent studies have shown that the molecular analysis of bile obtained from ERCP outperformed plasma analysis for CCA diagnosis.^[^ ^10^, ^16^ ^]^ For instance, next‐generation sequencing of cell‐free DNAs in bile showed a higher detection sensitivity for CCA, but the specificity remained below 70%. In contradistinctions, EVs are more abundant and stable and have been found in various body fluids such as ascites,^[^ ^17^ ^]^ bronchoalveolar lavage fluid,^[^ ^18^ ^]^ urine,^[^ ^19^ ^]^ bile,^[^ ^13^, ^14^, ^15^ ^]^ among others.

EVs are membrane‐bound nanovesicles actively shed by cells into circulation. Tumor‐derived EVs (tEVs) carry proteins and RNAs reflective of originating tumor cells,^[^ ^20^ ^]^ and thus serve as surrogate tumor markers. EV analyses have shown great promise in detecting cancers^[^ ^17^, ^21^, ^22^, ^23^, ^24^, ^25^, ^26^, ^27^ ^]^ and monitoring tumors’ responses to therapy.^[^ ^28^, ^29^, ^30^, ^31^, ^32^, ^33^ ^]^ Recent commercialization efforts accelerate the clinical translation of EV analysis in liquid biopsies. For example, the ExoDx Prostate Test, which analyzes EV RNAs from urine samples of patients with high PSA levels,^[^ ^34^ ^]^ received a breakthrough device designation from the U.S. Food and Drug Administration for ruling out unnecessary tissue biopsies. A number of clinical trials for EV biomarkers are ongoing.^[^ ^35^, ^36^ ^]^ However, EV biomarker studies for CCA are relatively limited compared to other cancer types. One study investigated EV concentrations in bile samples as a marker to discriminate patients with malignant from those with nonmalignant biliary stenosis.^[^ ^11^ ^]^ Because EVs are shed not only by tumor cells but also by host cells, EV counts alone can be non‐specific and have shown lower diagnostic powers in large cohort studies.^[^ ^21^, ^29^ ^]^ Therefore, detecting tEVs based on their molecular profiling of tumor biomarkers is critical.

Recent studies support single EV analysis technologies as the most promising option for early cancer detection.^[^ ^22^, ^26^, ^37^, ^38^ ^]^ This is because i) almost all types of cells shed EVs as background; ii) tEV amounts can be minuscule in small sizes of primary tumors; iii) not all tEVs contain tumor biomarkers. Single EV detection can improve our understanding of EVs’ various subtypes and heterogeneity and enables quantitative analysis. Several single EV sensing methods have been reported focusing mostly on technology development yet rather than analyses of primary human‐derived EV populations. Examples include nano‐flow cytometry,^[^ ^39^, ^40^ ^]^ interferometric imaging,^[^ ^41^, ^42^ ^]^ fluorescence EV imaging,^[^ ^22^, ^26^, ^43^ ^]^ plasmonic sensing,^[^ ^24^, ^44^ ^]^ and advanced microscopic techniques, such as fluorescence correlation spectroscopy^[^ ^45^ ^]^ and single‐molecule localization microscopy.^[^ ^46^ ^]^ A key component of single EV analysis remains robust signal amplification due to EVs’ weak signals associated with limited surface areas and epitopes available for immunolabeling. Among various signal amplification strategies, such as branched DNA probes^[^ ^47^ ^]^ or enzymatic reactions,^[^ ^22^ ^]^ plasmon‐enhanced fluorescence has shown robust fluorescence signal amplification across multiple channels.^[^ ^48^, ^49^ ^]^

Here, we report the development of an advanced plasmonic EV analysis technology for sensitive and robust single EV analysis. Termed “FLEX (fluorescence‐amplified extracellular vesicle sensing technology),” the technology harnesses plasmon‐enhanced fluorescence detection of EVs captured on periodic gold nanowell structures, enabling EV protein profiling at the single EV level using clinical samples. We applied FLEX technology to EV analyses in clinical bile samples from patients who underwent ERCP. The analysis showed that one could isolate and detect tEVs using key protein profiles in single EVs. Using the marker signature (MUC1, EGFR, EpCAM; EV^CCA^), we detected CCA with an area under the curve (AUC) of 0.93. These outcomes can lead to a well‐established liquid biopsy test for early CCA screening and detection.

## 2 Results and Discussion

### 2.1 FLEX Technology for Plasmon‐Enhanced Single EV Analysis

**Figure 1:**
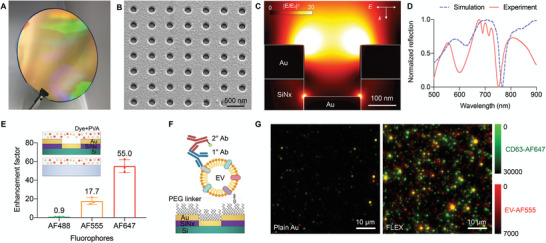
Fluorescence‐amplified extracellular vesicle (FLEX) sensing technology. A) FLEX gold nanowell chips fabricated on a 4 inch Si wafer. The fabricated wafer is diced into 1 cm^2^ chips for EV assays. B) A scanning electron micrograph of the FLEX chip shows the periodic gold nanowell structures. C) Finite‐difference time‐domain (FDTD) simulation shows the enhanced electromagnetic fields confined near the gold nanowell surface. The enhanced fields are responsible for plasmon‐enhanced fluorescence amplification. D) Reflectance spectra of the gold nanowell arrays with 200 nm diameter and 500 nm periodicity from FDTD simulation (blue dashed line) and experimental measurement (solid red line). E) Fluorescence signal enhancement of AF488, AF555, and AF647 fluorophores on the FLEX chips compared to a plain glass substrate. We formed a thin polyvinyl alcohol (PVA) layer containing the fluorescence dyes spun‐coated on the gold nanowell and glass surfaces, as shown in the schematic in the inset. The data are displayed as mean ± standard deviation from triplicate measurements. F) A representative schematic of an EV captured on the gold nanowell surface. The gold surface is functionalized by a polyethylene glycol (PEG) layer to capture EVs. The captured EVs are then labeled by primary (1°) antibodies followed by fluorophore‐conjugated secondary antibodies (2°). G) Representative fluorescence image of fluorescently labeled EVs captured on FLEX and plain gold substrates. For the same sample, EVs captured on the FLEX chip generate stronger fluorescence intensities.

A key concept of the FLEX technology is the ability to amplify signals from single EVs so that scarce biomarkers can be detected in rare tEVs. We designed the FLEX sensor with the following considerations: i) nanostructures are made in high‐throughput, ideally on a wafer‐scale through simple fabrication procedures; ii) the fluorescence enhancement range covers the typical EV size ranges (50–200 nm); iii) the resonance wavelengths can be readily tunable and reproducible. As such, we designed periodic gold nanowell structures with 200 nm well diameters and 500 nm periodicity made by interference lithography and metal deposition (**Figure** **1**A,B). The dimensions were determined by our previous work,^[^ ^17^, ^21^ ^]^ spectral overlap with commonly used fluorescence dyes, and reproducibility of nanowell fabrication. Interference lithography creates periodic nanostructures by exposing a photoresist layer on a Si wafer with two orthogonal grating beams.^[^ ^50^ ^]^ Subsequent reactive ion etching and gold deposition produce FLEX chips (see Experimental Section; Figure S1, Supporting Information, for detailed procedures). With wafer‐scale batch fabrication, we can construct a microarray‐type sensing array using a microarray spotter for high‐throughput analysis.^[^ ^21^ ^]^ The periodic nanowell structures support long‐range surface plasmon resonances extended to cover small EVs (e.g., exosomes) captured on a gold surface. The finite‐difference time‐domain (FDTD) simulation for nanowells with 200 nm diameter and 500 nm periodicity shows the strong field enhancement extended in a long‐range at a resonance wavelength, in addition to the localized enhancement along the nanowell edges (Figure 1C). The long‐range resonance induces homogenous signal enhancements for particles captured within the active area with periodic nanowell arrays, and the signal enhancement does not increase the coefficient of variances for particle intensities (Figure S2, Supporting Information). With our simple fabrication process, the optical properties of the fabricated FLEX chip were well‐matched with simulation results (Figure 1D). The resonance wavelengths can also be tuned by adjusting the periodicity of nanowell arrays.

To test the enhancement of fluorescence signals, we first formed a thin polyvinyl alcohol (PVA) layer containing fluorescence dyes (AF488, 555, and 647) on the nanowell and glass surfaces for comparison (Figure 1E). We achieved a 55‐fold signal enhancement with AF647 and a 17.7‐fold enhancement with AF555. The fluorescence signal enhancements are attributed to the spectral overlap between optical resonances of periodic nanowell structures and the excitation and emission spectra of AF555 and AF647 fluorophores (Figure S3, Supporting Information). In the case of AF488, which has optical spectra off of the plasmonic resonances, it shows comparable intensities between the two substrates. For EV detection, we first functionalized the gold surface with a mixture of 1 kDa carboxylated PEG and 0.2 kDa methylated PEG, which showed the optimal performance for EV capture.^[^ ^17^ ^]^ After EV capture, target EV proteins were labeled by primary antibodies followed by fluorescently labeled secondary antibodies (Figure 1F). Fluorescence images of EVs on the FLEX chip were imaged using a fluorescence microscope. To demonstrate that the signal enhancement was due to plasmonic resonances induced by periodic nanowell structures, we captured the same EV concentrations and fluorescently labeled them using the same procedures (Figure S4, Supporting Information). For the FLEX substrate, the underlying gold nanowell structures amplify EVs’ fluorescence signals both in AF555 (EV labeling) and AF647 (CD63 labeling) channels, which showed significantly higher intensities and detected EV counts compared to using a plain gold substrate (Figure 1G). The results showed that using FLEX chips as substrates could significantly improve EV detection sensitivity without requiring extra amplification processes or specialized instruments.

**Figure 2:**
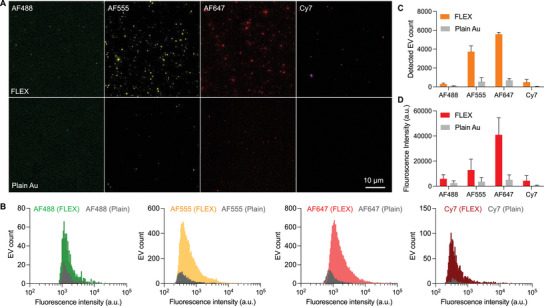
Characterization of plasmon‐enhanced EV detection with FLEX. A) Representative fluorescence images of EVs captured on FLEX and plain Au substrates. EVs were fluorescently labeled by AF488, AF555, AF647, and Cy7 fluorophores, respectively, and the same amount of EVs were captured on the gold surfaces using the same PEG linkers. B) Histograms of detected EV counts based on their fluorescence intensities. Colored bars represent EVs captured on FLEX chips, and gray bars represent EVs captured on plain Au substrates. C,D) Numbers C) and fluorescence intensities D) of detected EVs with FLEX and plain Au substrates. EVs were fluorescently labeled by AF488, AF555, AF647, and Cy7 fluorophores, respectively. The data are displayed as mean ± standard deviation from triplicate measurements

We next characterized the plasmon enhancements in different fluorescence channels for multiplexed EV analysis. We fluorescently labeled EVs from a cholangiocarcinoma cell line (SNU308) using tetrafluorophenyl (TFP) ester conjugated with AF488, AF555, AF647, or Cy7 dyes^[^ ^26^ ^]^(see Experimental Section for EV isolation and labeling protocols). We captured the fluorescently labeled EVs on the FLEX and plain gold substrates and compared the fluorescence intensities of individual EVs (**Figure** **2**). Here, we used plain gold substrates as a control for comparison in identical conditions. Although plasmon‐enhanced signal amplification is clearly seen with FLEX chips compared to glass substrates, as shown in Figure 1E and Figure S2 (Supporting Information), by using plain gold substrates, we could use the same material, thickness, surface chemistry, EV samples, and assay procedures for the comparison tests. As mentioned earlier, the Au surface was coated with 1 kDa PEG, which provides enough spacing between captured EVs and gold surfaces to prevent fluorescence quenching. Nonetheless, if there is any fluorescence quenching on a plain gold substrate, the same quenching would happen on the FLEX chip. Figure 2A shows representative EV fluorescence images of FLEX and plain substrates in the same color scale, and Figure 2B shows EVs’ intensity histograms. The results clearly show the fluorescence signal enhancements of EVs captured on the FLEX chip and significantly improved detection sensitivity in multiple channels. For EVs labeled by AF647, ≈5600 EVs were detected in 250 × 250 µm^2^ using the FLEX substrate, while less than 700 EVs were detected in the same‐sized area using the plain substrate. For EVs labeled by AF555, the detected EV counts were 3700 with the FLEX and 550 EVs with the plain substrate. As explained earlier, it should be noted that the same material, surface chemistry, and EV amounts were used for both substrates. The results indicate that conventional fluorescence imaging using a plain substrate detected only 10–15% of total EVs without a signal enhancement. This is likely due to weak fluorescence signals with small EVs; these weak signals get amplified on the FLEX chip by the underlying nanowell structures. In the case of EVs labeled by Cy7, a 16‐fold higher number of EVs (500 with nanowell vs 30 with plain) was detected with the FLEX substrate, but the absolute intensities were lower than EVs labeled by AF555 or AF647, which is likely due to the relatively weaker fluorescence signals of the NIR dye. Therefore, we determined to use AF555 for universal EV detection with TFP labeling and AF647 for CCA marker detection.

### 2.2 Single EV Analysis of CCA‐Derived EVs

To choose relevant CCA markers, we first reviewed the literature on biomarkers reported for CCA.^[^ ^51^, ^52^, ^53^, ^54^, ^55^, ^56^, ^57^, ^58^, ^59^, ^60^, ^61^ ^]^ We narrowed down the list based on the cross‐reference with the EV biomarker database (Vesiclepedia)^[^ ^62^ ^]^ and a human protein atlas.^[^ ^63^ ^]^ Moreover, we compared the candidate markers in our proteomics data of CCA cell lines with those in benign cells. This analysis led to the following candidate markers: EpCAM,^[^ ^53^ ^]^ EGFR,^[^ ^54^ ^]^ MUC1,^[^ ^51^, ^52^ ^]^ PD‐L1,^[^ ^55^ ^]^ WNT2,^[^ ^56^ ^]^ GPC1,^[^ ^57^ ^]^ and CD44v6.^[^ ^58^, ^59^ ^]^ We first performed cellular analysis on these candidate markers and EV putative markers (CD63, CD9, and CD81) using CCA cell lines (SNU308, SNU478, and SNU1196) and normal human cholangiocyte cell line (H69). The flow cytometry analysis showed that EpCAM, EGFR, and MUC1 are over‐expressed in CCA cell lines, while the levels were negligible in the control H69 cell line (Figure S5, Supporting Information). The over‐expressions of EpCAM, EGFR, and MUC1 in CCA were also validated by conventional immunohistochemistry (IHC) staining of tissue samples from CCA patients (Figure S6, Supporting Information) and proteomic analyses (Figure S7, Supporting Information).

**Figure 3:**
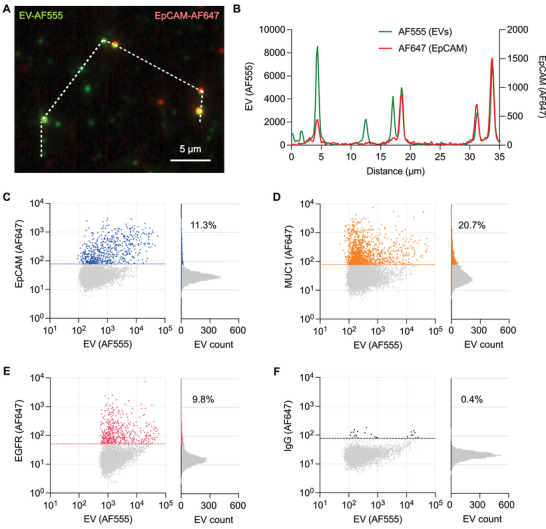
Single CCA EV analysis with FLEX. A) Representative two‐channel fluorescence images of SNU308 EVs. All captured EVs are labeled by AF555 (green), and EpCAM was detected by immunolabeling with anti‐EpCAM antibody followed by AF647 secondary antibodies (red). B) Fluorescence intensity line profiles in AF555 (green) and AF647 (red) channels along the white dashed line shown in (A). AF555 represents EV signals, and AF647 represents EpCAM signals. C–F) 2D scattering plots of EVs from a SNU308 cell line. The x and y axes represent AF555 (universal EV staining) and AF647 (cancer biomarker) intensities, respectively. When AF555 and AF647 intensities are colocalized, EVs are counted. The graphs on the right side represented the colocalization ratios of EpCAM, MUC1, EGFR, and IgG (isotype control). The marker threshold value dashed lines along the *y*‐axis were defined by mean + 3 × standard deviation of IgG signals

We then measured the three markers across EVs derived from CCA cell lines and investigated their heterogeneity from single EV analyses with FLEX. After EV isolation from cell culture supernatants, isolated EVs were labeled by TFP‐AF555. Then, ≈5 × 10^5^ EVs diluted in PBS were captured on the FLEX chip surface following NHS/EDC activation. The captured EVs were then immunolabeled with primary antibodies for target cancer and EV markers (EpCAM, MUC1, EGFR, and CD63), followed by labeling with AF647‐conjugated secondary antibodies. **Figure** **3**A,B shows a representative fluorescence image and corresponding intensity profiles of SNU308‐derived EVs in the TFP (AF555) and marker (AF647) channels. In the analysis, we first located EV positions in the AF555 channel by detecting spots with a signal‐to‐noise ratio (SNR) greater than 3. At each detected EV position, we measured the cancer marker intensity in the AF647 channel. In this way, we could minimize false‐positive marker signals due to the nonspecific binding of antibodies to the sensing surface. Furthermore, we set the marker positivity threshold using an IgG control where the threshold value was defined by the mean + 3 × standard deviation of IgG signals. For each marker, we obtained 4 images in which the field of view of each image was ≈250 × 250 µm^2^ covered by 1 µl EV solution. On average, we detected and analyzed ≈7437 ± 636 (*n* = 27) EVs per field of view. For the same amount of EVs applied, we can see significantly higher numbers of TFP‐ and CD63‐positive EVs using the FLEX substrate than a plain substrate (Figure S8, Supporting Information). Interestingly, for each of the three tumor markers (EpCAM, EGFR, and MUC1), only 10–20% of total EVs contain all tumor markers with a wide range of expression levels and a more than one‐order difference (Figure 3C–F). In contrast, all three markers are present in the CCA cell line‐derived EVs with significantly higher ratios than an IgG control (*p* < 0.001, one‐way ANOVA with Dunnett's multiple‐comparison test, Figure S9, Supporting Information). The false‐positive rate in the IgG control channel was measured at less than 0.5%. The marker positivity rates substantially increased for EV subpopulations with strong TFP signals (Figure S10, Supporting Information). For example, the rate of MUC1‐positive EVs increased to 33% for EVs with TFP‐AF555 signals greater than 10^3^ and 74% for EVs with TFP‐AF555 signals >10^4^. However, with no thresholds, 33‐ and sixfold higher numbers of MUC1‐positive tEVs were detected compared to the 10^4^ and 10^3^ threshold cases, respectively. The TFP signal intensities, in general, correlate with dark‐field scattering light intensities of EVs (Figure S11, Supporting Information). This indicates that most EVs were in small sizes (weak fluorescence intensity), which could be missed without signal amplification. Without the enhancement, it could also provide biased analysis by analyzing larger (or aggregated) EVs with strong signals. These differences can be critical for early cancer detection, where tEV counts are relatively low. The assay also shows good reproducibility with a chip‐to‐chip coefficient variation below 5% (Figure S12, Supporting Information).

**Figure 4:**
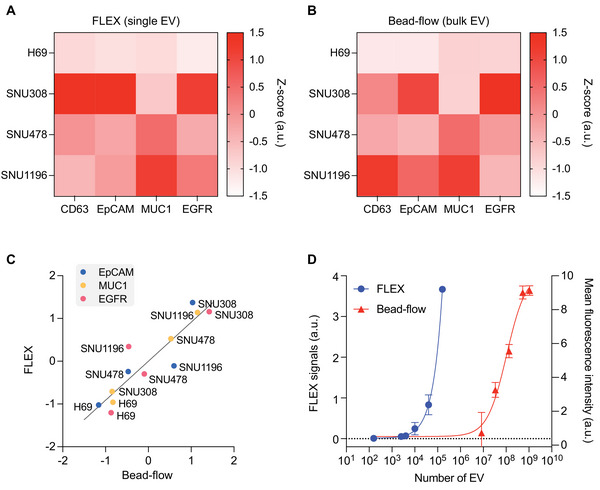
EV protein profiling with FLEX. A,B) CCA (EpCAM, MUC1, and EGFR) and EV (CD63) markers were profiled for EVs from three different CCA cell lines and one normal cell line by single EV analysis with FLEX A) and bulk EV analysis with bead‐flow assays B). C) Correlation of z‐scores for each marker between FLEX and bead‐flow measurements. The Pearson correlation coefficient *r* = 0.91 (*P* < 0.0001). D) Comparison of the detection sensitivity of FLEX and bead‐flow assays. Aliquots of samples in the same volume (30 µl) were used for direct comparison. The detection limits were determined by titrating known EV quantities and measuring their CD63 signals. The data are displayed as mean ± standard deviation from duplicate measurements.

We extended the analysis to other cell line‐derived EVs and compared the results with bulk EV analysis using bead‐based flow cytometry (**Figure** **4**A,B). We chose bead‐based flow cytometry, as the method showed higher sensitivity than other bulk EV methods (ELISA and Western blot)^[^ ^64^ ^]^ and can be run with a similar configuration to the FLEX assay; EVs were immobilized on the bead surface, followed by labeling using the same sets of primary and fluorescent secondary antibodies (Table S1, Supporting Information). The heatmaps showed the z‐scores of normalized marker levels compared to isotype controls. Linear regression analysis showed a good linear correlation (Pearson *r* = 0.91) between the single EV analysis and the bulk EV approach using flow cytometry (Figure 4C). The bead‐based bulk EV analysis with flow cytometry achieved a limit of detection of 7.5 × 10^7^ EVs, whereas the FLEX technology achieved a limit of detection of 2 × 10^3^ EVs. (Figure 4D). Here, we used the aliquots of samples in the same volume (30 µl) for direct comparison. The limit of detection indicates the number of EVs required for reliable detection to determine marker positivity. This is mainly limited by the diffusion of EVs to the sensor surface for capture. Thus, we expect a lower required amount by reducing the diffusion time. Applying a microfluidic channel with herringbone patterns^[^ ^31^ ^]^ or field‐induced mixing^[^ ^65^ ^]^ could potentially reduce the diffusion time, leading to a lower detection limit. Combined with plasmon enhancement, the 4‐orders of magnitude increase in sensitivity could enable the detection of rare tumor‐derived EVs in clinical samples and their protein markers.

### 2.3 EV Analysis of Clinical Samples for CCA Diagnostics

**Figure 5:**
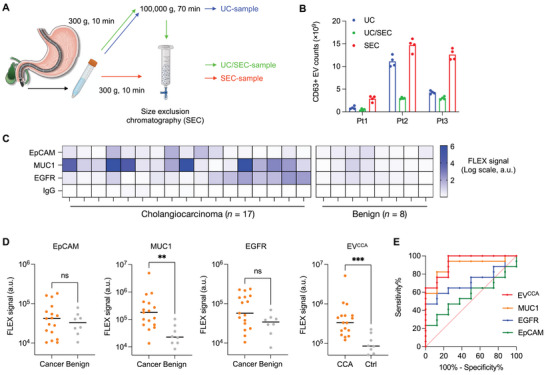
Profiling of CCA patient EVs with FLEX. A) EV isolation from human bile samples. We tested ultracentrifugation (UC), size exclusion chromatography (SEC), and their combination for EV isolation. B) Comparison of CD63‐positive EV counts. EVs were isolated from three patient bile samples using methods depicted in (A). SEC method showed the highest CD63‐positive EV counts for all three samples tested. The data are displayed as mean ± standard deviation from quadruple measurements. C) Analysis of bile‐derived EVs from 17 CCA and 8 benign patients for selected CCA markers (EpCAM, MU1, and EGFR). IgG isotype was used as a negative control. The FLEX signals from each marker are represented in a heatmap on a log scale. D) FLEX signals on each tEV marker (EpCAM, MUC1, and EGFR) and their combination (EV^CCA^) as measured in bile‐derived EVs from 17 CCA and 8 benign patients. Mann–Whitney unpaired *t*‐test was done (**P < 0.01 and ***P < 0.001). E) Receiver operating characteristic curves of each tEV marker and intensity and EV^CCA^. The EV^CCA^ signature showed the highest area‐under‐the‐curves (AUC) value of 0.93.

We next applied the FLEX assay in a pilot study to detect CCA‐derived EVs using clinical samples. We collected bile samples (4–14 mL, mean = 6.8 mL) from CCA (*n* = 17) and non‐cancer patients (*n* = 8) by ERCP. We first tested different EV isolation methods for bile samples, including the gold standard ultracentrifugation, size‐exclusion chromatography (SEC), and their combination. Ultracentrifugation (UC) is a preferred method for a large volume of samples with a high isolation capacity. On the other hand, SEC isolates EVs based on their unique size range, larger than soluble proteins and smaller than cell debris. After removing floating dead cells and debris by centrifugation at 300 g, we isolated EVs from 1 mL of bile samples (*n* = 3) using the three methods (**Figure** **5**A) and evaluated CD63‐positive EV counts, total EV counts, and protein levels (Figure 5B; Figure S13, Supporting Information). SEC showed better EV isolation efficiency in total and CD63‐positive EV counts than UC and UC/SEC combination. Interestingly, the protein amounts per EV were higher in UC than in SEC. This result may represent the major weakness of UC isolation for protein contamination in isolated EVs. The UC/SEC combination showed fivefold lower CD63‐positive EV counts than using SEC only. This can imply that the combination method led to a significant loss of EVs through the two‐step process. Based on these results, we decided to use SEC for EV isolation from bile samples.

We expanded the FLEX analysis to 25 patients (*n* = 17 for BTC patients and *n* = 8 for benign patients) using EpCAM, MUC1, and EGFR (Figure 5C). The clinical details of these patients are described in **Table** **1**. While a previous study showed elevated EV counts in CCA patients,^[^ ^15^ ^]^ in this cohort, we do not see a significant difference in total EV counts between the two groups (Figure S14, Supporting Information). However, we can see a different differential pattern from marker‐positive tEVs. Here, we detected tEVs based on marker positivity and measured their counts and individual EVs’ intensities (Figure S15, Supporting Information). For FLEX signals, we calculated the total fluorescence intensities of marker‐positive EVs to take into account both the tEV count and their marker levels. For marker‐positive EVs, MUC1 showed significantly elevated signals from CCA patients compared to benign patients (*P* = 0.001 for MUC1, Mann–Whitney unpaired *t*‐test. Figure 5D), but the area under the curve (AUC) remained below 0.9. While EpCAM and EGFR do not show significant increases in EVs from CCA patients as single markers, their combinations with MUC1 (we termed “EV^CCA^”) further increased the difference between CCA and benign groups (*P* = 0.0003, Mann–Whitney unpaired *t*‐test) and increased the AUC to 0.93 (Figure 5E). This is because adding EGFR and EpCAM allowed us to detect cases with low or no MUC1 expressions. The AUC of CA19‐9 was only 0.69, and the diagnostic accuracy with a clinical threshold value of 37 U mL^−1^ was 74% (17/23), similar to previously reported ranges (Figure S16, Supporting Information).^[^ ^5^, ^6^ ^]^ Among the six patients with abnormal CA 19‐9 values, five were correctly discriminated using EV^CCA^. EVs are attractive circulating biomarkers with their abundance, stability, and molecular cargos from originating cells. Namely, molecular analysis of tumor‐derived EVs in biofluids can offer a liquid biopsy‐based molecular diagnosis of cancer. We developed the FLEX technology to better facilitate tumor‐derived EV detection and molecular analysis that can be integrated into the clinical workflow. The FLEX technology significantly improves the EV detection sensitivity using plasmonic enhancements of fluorescence signals with periodic gold nanowell structures. For the same EV aliquots, the FLEX chip detected an eightfold higher number of EVs than conventional plain substrates. This indicates that ≈90% of EVs could be missed by traditional immunofluorescence labeling and imaging. This is why the EV sensing field has focused on different signal amplification strategies for sensitive EV detection. However, our FLEX approach achieved high sensitivity to the single EV level without requiring specialized instruments or other signal amplification processes. The high sensitivity is particularly crucial to detect rare EV targets (e.g., scarce tEVs from small sizes of tumors at early stages, phosphorylated or mutated proteins in EVs^[^ ^26^ ^]^). The cancer‐specific mutation marker detection in tEVs could help improve the diagnosis specificity. Furthermore, as shown in Figure 2, the enhancement could occur in multiple channels (AF555, AF647, and Cy7), boosting the sensitivity compared to conventional fluorescence imaging. It is because the periodic nanowell structure supports multiple resonances in different wavelengths. While we did not fully take advantage yet in this work, the plasmonic enhancement would be helpful to extend the multiplexing capability in NIR channels that are barely used in conventional EV fluorescence imaging due to weak signals. This could lead to a higher degree of multiplexing.

**Table 1:** Clinical information of patients

**Characteristic**	**Cholangiocarcinoma [*n* = 17]**	**Non‐maligant obstruction [*n* = 8]**
**Primary location of maligancy [%]**		
Perihilar bile duct	5 (29.4)	–
Distal bile duct	12 (70.6)	–
**Cause of biliary obstruction [%]**		
Biliary stones	–	7 (87.5)
Benign biliary stricture	–	1 (12.5)
**Age, yr**		
Median (range)	71 (37–80)	67.5 (50–80)
**Sex [%]**		
Male	10 (58.8)	4 (50)
Female	7 (41.2)	4 (50)
**Bile sample volume [mL]**		
Mean (min, max)	6.9 (4, 14)	6.4 (5, 8.5)
**CA19‐9, U mL^−1^**		
Mean (SD)	1606.5 (4776.7)	336.6 (777.2)
**Total bilirubin, mg dL^−1^**		
Mean (SD)	7.5 (5.7)	3.4 (3.1)
**CRP, mg L^−1^**		
Mean (SD)	30.1 (26.8)	40.0 (42.0)
**Stage of tumor [%]**		
I	3 (17.6)	–
II	8 (47.1)	–
III	0 (0.0)	–
IV	6 (35.3)	–
**Pathological confirmation methods [%]**		
1st ERCP sampling	12 (70.6)	–
2nd ERCP sampling	1 (5.9)	–
Surgery	2 (11.8)	–
Other method^a)^	2 (11.8)	–

Abbreviation: SD, standard deviation; ERCP, endoscopic retrograde cholangiopancreatography.

^a)^ Liver biopsy and ascites cytology were used.

During the development of FLEX technology, we focused on the simple, robust, and wafer‐scale fabrication of FLEX chips, which is a key component to achieving higher detection sensitivity with good reproducibility. The low‐cost, high‐throughput chip fabrication often becomes a critical bottleneck when translating highly sensitive plasmonic sensing technologies into clinical applications. We could produce periodic nanowell structures in 4 inch Si wafers using interference lithography and metal evaporation. The current process costs ≈$750 per wafer or $12.5 per 10 by 10 mm^2^ chip. We expect the cost will significantly scale downward with bulk production. Alternatively, nanoimprint or deep ultraviolet (DUV) lithography could be used to pattern periodic nanowells on a wafer scale. Using DUV lithography could provide us more flexibility to further optimize nanowell structures, sizes, and periodicity to maximize the field enhancements and enable a higher degree of multiplexing. The high‐throughput, low‐cost chip fabrication will open up opportunities to adapt the signal enhancement in other advanced microscopic techniques.^[^ ^45^, ^46^ ^]^

CCA is a highly heterogeneous malignancy with multiple subtypes based on its anatomical location along the biliary tree. Therefore, we set out to identify a marker combination rather than relying on single markers for CCA detection. Through bioinformatic survey and cross‐references with EV and protein marker databases, we narrowed candidates to 10 markers, including 7 tumor‐associated (EpCAM, EGFR, MUC1, PD‐L1, WNT2, GPC1, and CD44v6) and 3 EV putative markers (CD63, CD81, and CD9). Through proteomics and flow cytometry analysis on tissues, cells, and EVs, we constituted the diagnostic marker EV^CCA^ signature (MUC1, EpCAM, and EGFR). Applying the EV^CCA^ signature to bile samples from 25 patients, the FLEX EV analysis showed high classification accuracy (AUC = 93%), which could be attributed to the release of tEV in bile samples and FLEX's high sensitivity in detecting tEVs from a vast background of non‐tumor EVs. It should be noted that the sensitivity of an ERCP sampling with conventional clinical pathology remained at 70% (Table 1); the other 30% required additional procedures for confirmation. The EV analysis of bile samples obtained during ERCP procedures could be an excellent complementary test to reduce non‐diagnostic cases.

The current study had some limitations that need to be addressed in future studies. As a feasibility test, it is encouraging that all three patients in Stage I were correctly detected by the FLEX analysis. A more extensive cohort study needs to be conducted for a formal statistical analysis based on cancer stages. Testing for large cohorts in prospective trials will also establish the clinical utility of FLEX analyses. Expanding the marker panel to mutated oncoproteins (e.g., KRAS mutations) or tumor suppressor proteins could further improve the detection accuracy, especially for early cancer detection.^[^ ^26^ ^]^ Multiplexed analysis of both EV proteins and RNA markers could provide more comprehensive analyses and find broader applications.

As a next step, we plan to expand the current study to blood samples. Compared to other cancer types, there are few studies on CCA detection through EV analysis. Therefore, the current bile EV analysis was important to identify the presence of tumor‐derived EVs in biofluids, analyze their protein signatures, and the correlation with originating tumors. Although the current study was not designed for blood samples, we have conducted such clinical studies for other cancer types, including pancreatic cancer.^[^ ^17^, ^21^, ^26^, ^64^ ^]^ Notably, it could allow us to serially assess patients and enable treatment monitoring. The sensitive single EV analysis using FLEX could facilitate the detection of scarce tumor‐derived EVs and quantify their temporal changes in cancer development and therapy responses.

## 3 Experimental Section

### Clinical Sample Collection

Bile samples were prospectively collected at the Severance Hospital, Yonsei University College of Medicine, Seoul, Korea, from 26 patients undergoing ERCP due to biliary obstruction. One bile sample was excluded from the analysis due to a large amount of impurities and large aggregates during EV isolation. Among the 25 samples analyzed, 17 patients were diagnosed with cholangiocarcinoma, and 8 patients were diagnosed with benign biliary obstruction. All ERCP procedures with therapeutic video duodenoscopy (TJF‐260 V) were performed by interventional endoscopists with experience in at least 1000 cases. Experienced attending anesthesiologists sedated all the patients. After selective biliary cannulation, 5–10 mL of bile was aspirated via a biliary catheter. After acquiring a cholangiogram to evaluate the biliary obstruction, tissue acquisition was performed by using intraductal biopsy if malignancy was suspected. Diagnosis of cancer was established by the following methods in all cases: i) surgical pathology; ii) pathologic diagnosis made by ERCP tissue biopsy with evidence for malignancy; and iii) pathologic diagnosis made by other tissue acquisition methods, such as percutaneous biopsy or endoscopic biopsy for metastasis or direct invasion of the tumor to other organs. For patients with benign conditions, such as choledocholithiasis, the patients were followed up for at least 1 year without evidence of malignancy. The protocol of the present study adhered to the Declaration of Helsinki and was approved by the Institutional Review Board of Severance Hospital (IRB number: 4‐2018‐1115) and Massachusetts General Hospital. Written informed consent was obtained from all subjects.

### EV Isolation from Bile Samples

Bile sample (1 mL) from each patient was centrifuged with 300 x g for 10 min at 4 °C to remove floating cells or large debris. Then, the bile sample was centrifuged with 2000 x g for 20 min at 4 °C to remove larger particles, such as apoptotic bodies. EVs were isolated using 3 methods (ultracentrifugation (UC), size‐exclusion chromatography (SEC), and a combination of UC and SEC) and the better methods were compared. For EV isolation using UC, the sample was centrifuged 100 000 x g for 70 min at 4 °C with a polypropylene tube for SW 32.1 Ti rotor of Optima™ Ultracentrifuge (Beckman Coulter). Then, the pellet was washed with PBS and centrifuged again with 100 000 x g for 70 min at 4 °C. The EV pellet was resuspended in PBS. For EV isolation using SEC, first an SEC column was prepared with Sepharose CL‐4B (GE Healthcare) based on the previously published protocol.^[^ ^66^ ^]^ Briefly, An 11 µm pore‐sized nylon membrane (NY1102500, Millipore Sigma) was placed on the bottom of a 10 mL syringe (BD Biosciences). The syringe was stacked with 10 mL of Sepharose and triple‐washed with PBS. Then, the bile sample was applied, and the 4th and 5th fractions (1 fraction = 1 mL) were collected for EVs. The collected sample was concentrated using Amicon Ultra‐2 Centrifugal Filter (MWCO = 10 kDa, Millipore Sigma) and centrifuged at 3500 x g for 30 min at 4 °C. The isolated EVs were resuspended in PBS. For EV isolation using a combination of UC and SEC, EVs were first isolated by UC with 100 000 x g for 70 min at 4 °C. The pellet was diluted in 1 mL of PBS and passed through an SEC column. The 4th and 5th fractions were collected and centrifuged at 3500 x g for 30 min at 4 °C with an Amicon Ultra‐2 Centrifugal Filter (MWCO = 10 kDa, Millipore Sigma). The isolated EVs were resuspended in PBS. The isolated EVs were fluorescently labeled using the published protocol.^[^ ^26^ ^]^ Briefly, EVs were mixed with 0.2 µl TFP‐AF555, followed by 1 h of incubation. The labeled EV was filtered with a 40 K MWCO column (Thermo Fisher) to remove the remaining dye and was diluted in PBS before the assay.

### Cell Lines

SNU308, SNU478, and SNU1196 were provided by Yonsei University. SNU308, SNU478, and SNU1196 were grown in Roswell Park Memorial Institute (RPMI) 1640 medium (Gibco) supplemented with 10% fetal bovine serum (FBS, Thermo Fisher), 100 U mL^−1^ penicillin, and 100 µg mL^−1^ streptomycin (Gibco) at 37 °C in 5% CO2. H69 cells were generously provided by Prof. Yangmi Kim and Prof. Seon Mee Park at Chungbuk National University College of Medicine. H69 cells were maintained in enriched Dulbecco's minimum essential medium (DMEM) (Hyclone) containing 10% fetal bovine serum (FBS) (Gibco, Invitrogen), 0.025 mg ml^−1^ adenine (Sigma, St. Louis, Mo, USA), 0.005 mg ml^−1^ insulin (Gibco Invitrogen), 0.002 mg ml^−1^ epinephrine (sigma), 13.6 ng ml^−1^ T3T triiodo_L_thyronine (T3) (sigma), 0.0083 mg ml^−1^ holo‐transferrin (Gibco, Invitrogen). Hydrocortisone (Sigma) (620 ng ml^−1^) and 10 mg ml^−1^ epidermal growth factor (EGF; CytoLab Ltd., Rehovot, Israel) at 37 °C in 5% CO_2_. All cell lines were tested and free from mycoplasma contamination (Universal Mycoplasma Detection Kit, ATCC).

### EV Isolation from Cell Lines

Cells were incubated in a medium with 2% exosome‐depleted FBS (Thermo Fisher) for 48 h, followed by EV collection. The conditioned medium was collected through a cell strainer (40 µm Nylon, Thermo Fisher) and filtered through a 0.2 µm membrane filter (Millipore Sigma). The conditioned medium was concentrated with Centricon Plus‐70 Centrifugal Filter (MWCO = 10 kDa, Millipore Sigma) and centrifuged at 3,500 g for 30 min at 4 °C. The concentrated medium was passed through with SEC. Similar to EV isolation from bile samples, the 4th and 5th fractions were used for EV isolation, followed by concentration using Amicon Ultra‐2 Centrifugal Filter (MWCO = 10 kDa, Millipore Sigma) and centrifuged at 3500 x g for 30 min at 4 °C. The isolated EVs were reconstituted in PBS, aliquoted, and stored in a −80 °C deep freezer. Total EV protein was measured using a Qubit assay kit (ThermoFisher, Q33212). The isolated EVs were characterized by transmission microscopy, western blot, and nanoparticle tracking analysis (Figure S17, Supporting Information).

### FLEX Chip Fabrication

First periodic nanowell arrays were patterned (200 nm in diameter and 500 nm in periodicity) using interference lithography, which was done through LumArray, Inc (Somerville, MA, USA). Briefly, a 200 nm thick, low‐stress silicon nitride layer was first deposited by low‐pressure chemical vapor deposition (LPCVD) on 4 inch Si wafers. After anti‐reflection coating (ARC) and spin‐coating of a negative photoresist, two orthogonal grating images were exposed to the photoresist and made periodic nanowell patterns. Subsequent reactive ion etching with CF_4_ transferred the hole patterns into the silicon nitride layer. The remaining resists were removed by piranha cleaning.^[^ ^67^ ^]^ Deposition of 100 nm thick Au with a 5 nm Ti adhesion layer produced periodic Au nanowell arrays. The wafers were then diced into smaller pieces for EV assays.

### FLEX EV Assay

FLEX chips were serially cleaned with acetone, IPA, and deionized water. The sensor chip surface was functionalized with a mixture of SH‐PEG‐COOH 1 k (Nanocs) and SH‐mPEG 0.35 k at a ratio of 1:3 overnight. For EV capture, the gold surface was incubated in 0.2 m EDC (Thermo) and 0.05 m sulfo‐NHS (Thermo) for 7 min to capture EVs by covalent bonding. After gently washing with PBS, EVs were introduced to the sensor chip and incubated for 30 min. After EV capture, EVs were fixed by 4% paraformaldehyde for 10 min, followed by blocking with 2% BSA for 20 min. The captured EV were immuno‐fluorescently labeled by primary antibodies for 60 min, followed by secondary antibody (AlexaFluor 647 anti‐mouse) incubation for 30 min. Antibodies were diluted in 0.2% BSA solution, and staining was performed under agitation. Each antibody was diluted with its dilution factor, which was determined by screening optimal antibody concentrations (CD63:1/100, EpCAM:1/20, MUC1:1/800, EGFR:1:20). Finally, the chips were mounted with a mounting solution (ProLong Gold Antifade mountant, Thermo Fisher) and covered with a glass coverslip. Fluorescence images were acquired on Nikon Ti inverted automated epifluorescence microscope with a 40 × (NA = 0.95) objective lens.

### Image Processing

Images were analyzed using ImageJ (ImageJ2 Fiji, version 2.3.0/1.53q) and custom‐built MATLAB (version R2015a) code. Image shift between fluorescence channels was registered and corrected using the ImageJ NanoJ plugin. Then background signals were subtracted using a rolling ball algorithm (radius = 50). Then the ComDet plugin was used in ImageJ to detect EV locations using AF555 signals. Although the ComDet plugin also provides fluorescence intensities of individual detected particles from a dynamic pixel window, the authors found that the dynamic method sometimes overestimated intensity values when particles form dimers or larger aggregates (Figure S18, Supporting Information). Instead, averaged fluorescence intensities were calculated from a 3 × 3 fixed pixel window in AF647 channels.

### Flow Cytometry Analysis for Cell Lines

SNU308, SNU478, SNU1196, and H69 cells were trypsinized, washed with PBS twice, and then fixed with chilled 2% paraformaldehyde for 1 h. Cells were blocked by 0.5% BSA and 2% normal fetal bovine serum on ice for 30 min. The cells were then incubated with primary antibodies and washed with PBS, followed by secondary antibody labeling with Alexa Fluor 488. The flow cytometry measurements were obtained with a BD FACS LSR II SORP system, and the data were analyzed using Flow Jo software (version 10.2). The excitation beam for the GFP was set at 488 nm, and the emission signal was captured with a 525 50 nm^−1^ bandpass filter. The gain voltages were set by default to 625, 420, and 600 V for forward scatter (FSC), side scatter (SSC), and GFP acquisition, respectively, and events were created for measurements where FSC > 200 & SSC > 200.

### Flow Cytometry for EVs

EVs (10^9^ and 10^10^) were incubated with 0.2 µL aldehyde/sulfate beads (Invitrogen, A37304) for 30 min to saturate the beads with EVs, followed by blocking with 1% BSA in PBS for 2 h. After coating with glycine, beads were washed with PBS using centrifugation. Then, beads were incubated with primary antibodies diluted to 10 µg mL^−1^ in PBS with 1% BSA for 1 h, followed by another 1 h incubation with secondary antibodies. Samples were analyzed with a CytoFlex flow cytometer (Beckman Coulter, A00‐1‐1102) using the 488/8 and 525/40 nm bandpass filter for SSC and FITC, respectively, and the following settings (FSC 201 V, SSC 90 V, FITC159V). FlowJo X 10.0 was used for analyzing median fluorescence intensity. For normalization, the median fluorescence intensity was divided with the signal of the isotope control. Moreover, the z‐score was calculated using each marker's mean value and standard deviation.

### Transmission Electron Microscopy

For sample preparation, a drop of sample was placed on the Formvar‐carbon coated grid for 15 s, the droplet was removed using filter paper, a drop of 1% uranyl acetate was put for 15 s, and removed using filter paper, and washed with a drop of distilled water. Dried grids were imaged with transmission electron microscopy (JEM‐1011, JEOL, Tokyo, Japan) at the acceleration voltage of 80 kv equipped with a Megaview III CCD camera (Softimaging system‐Germany).

### Immunohistochemistry

Immunohistochemistry (IHC) was done on paraffin‐embedded tissue with Mucin 1 (MUC1), epithelial cell adhesion molecule (EpCAM), and epidermal growth factor receptor (EGFR) using the standard immunohistochemistry technique. IHC staining was done manually after antigen retrieval by boiling slides in 10 mm sodium citrate buffered distilled water (pH 6.0) for 20 min in a 97 °C water bath, followed by a 30 min cooldown period. Primary antibodies used were monoclonal anti‐MUC1 antibody (catalog no. 10‐M93B) and anti‐EpCAM antibody (catalog no. ab20160) at a dilution of 1:200 and anti‐EGFR antibody (catalog no. sc365829) at a dilution of 1:100. Primary antibodies were incubated for 19 h at 4 °C. The Dako REAL Peroxidase Detection System Kit was used according to the manufacturer's instructions, including the ready‐to‐use‐anti‐rabbit/mouse secondary antibody (catalog no. K5007) and counterstained with hematoxylin solution (catalog no. 03 971)

### Mass Spectrometry Analysis

EV samples were analyzed using an LC‐MS/MS system consisting of an UltiMate 3000 RSLCnano system (Thermo Fisher Scientific) and an Orbitrap Eclipse Tribrid mass spectrometer (Thermo Fisher Scientific) equipped with a nano‐electrospray source (EASY‐Spray Sources, Thermo Fisher Scientific). Please see Supporting Information for sample preparation. Peptides from EV samples were trapped 75 µm × 2 cm C18 pre‐column (nanoViper, Acclaim PepMap100, Thermo Fisher Scientific) before being separated on an analytical C18 column (75 µm × 50 cm PepMap RSLC, Thermo Fisher Scientific) at a flow rate of 250 nL min^−1^ and total run time of 70 min. Mobile phases A and B comprised 100% water containing 0.1% formic acid and 100% acetonitrile containing 0.1% formic acid, respectively. The voltage applied to produce an electrospray was 2,000 V. During the chromatographic separation, the Orbitrap mass spectrometer was operated in data‐dependent mode, automatically switching between MS1 and MS2. The MS data were acquired using the following parameters: Full scan MS1 spectral (400–2000 m z^−1^) were obtained in the Orbitrap for a maximum ion injection time of 50 ms at a resolution of 120,000 and a standard mode automatic gain control (AGC) target. MS2 spectra were acquired in the Orbitrap mass analyzer at a resolution of 30,000 with turbo‐TMT setting applying high energy collision dissociation of 36% normalized collision energy and AGC target value of 5.0 × 10^4^ with a maximum ion injection time of 54 ms. Previously fragmented ions were excluded for 30 s.

### Proteome Search and Bioinformatics Analysis

A proteome search was performed based on the previously published protocol^[^ ^68^ ^]^ with minor modifications as followed. The MS raw files were converted into mzML using MSConvert (version 3.0.20033). Ms2 files were extracted from the mzML using an in‐house program coded by Python 3.8 from Anaconda 3 environment (version 3.8.0). To analyze the proteins in EVs, a proteome database was generated from Uniprot and Integrated Proteomics Pipeline version 5.1.2. (IP2, Integrated Proteomics Applications Inc., San Diego, CA). Proteome search results were evaluated by the false discovery rate at spectra and protein level with less than 1.0% using DTASelect (Integrated Proteomics Applications Inc., San Diego, CA), respectively. Protein quantification for the discovery of DEP was performed from the ms2 files with TMT reporter ions using the Census software (Integrated Proteomics Applications Inc., San Diego, CA). Each data was normalized with H69 data to show the fold change in cholangiocarcinoma cell lines and EVs over those in the H69 cell line and EVs.

### Nanoparticle Tracking Analysis

The number of EVs was measured by nanoparticle tracking analysis (Nanosight LM10 microscope, Malvern). The experiment was conducted with a 642 nm laser module at room temperature. Each sample was diluted 500‐fold in PBS and manually placed in the chamber. Each experiment was performed for 30 s in quadruplicate. The number of particles per frame was 33.3–87.7, and the frame rate per second was 30.

### Statistical Analysis

The data were analyzed with GraphPad Prism version 9 (GraphPad Software Inc., San Diego, CA, USA). All data were displayed as mean ± standard deviation. The Mann–Whitney unpaired *t*‐test was used to compare two independent groups. For comparing more than two groups, a one‐way ANOVA test was used. Statistical significance was accepted for values of *p* < 0.05.

## Conflict of Interest

H.I. is a consultant to Aikili Biosystems, Noul, and Cellkey and receives research support from Canon USA, CytoGen, Healcerion, and Noul. R.W. is a consultant to ModeRNA, Lumicell, Seer, Earli, and Accure Health. C.M.C. is a consultant to Aikili Biosystems, Qiagen, Teladoc, and InfiniteMD. The remainder of the authors reports no industrial interactions.

## Author Contributions

M.H.J., T.S., and Y.K.T. contributed equally to this work. H.I., S.B., and J.H.J. conceived the idea and designed the study. M.H.J. and T.S. developed the FLEX technology and assay. M.H.J., T.S., Y.K.T., and C.H.P. performed the research. H.S.L., M.J.C., J.Y.P., J.H.J, and S.B. collected clinical samples. M.H.J. and Y.K.T. processed clinical samples. C.M.C. and R.W. assisted in the clinical study design. M.H.J., T.S., Y.K.T., C.M.C., R.W., J.H.J., and H.I. analyzed the results. M.H.J., T.S., Y.K.T., J.H.J., and H.I. wrote the manuscript, edited by all authors.

## Supporting information

Supporting Information

Supporting Information

Supporting Information

Supporting Information

Supporting Information

## Data Availability

The data that support the findings of this study are available from the corresponding author upon reasonable request.
