# Supplementary material for: Plasmon‐Enhanced Single Extracellular Vesicle Analysis for Cholangiocarcinoma Diagnosis
Source: Adv Sci (Weinh). 2023 Jan 25;10(8):2205148. doi: 10.1002/advs.202205148 (PMC10015870; doi:10.1002/advs.202205148)
Supplement: Supplementary file 1 — Supporting Information [file ADVS-10-2205148-s004.pdf]

Supporting Information

Plasmon-enhanced single extracellular vesicle analysis for cholangiocarcinoma diagnosis

Mi Ho Jeong[1†], Taehwang Son[1†], Yoo Keung Tae[2†], Hee Seung Lee[2], Moon Jae Chung[2], Jeong Youp Park[2], Cesar M. Castro[1,3], Ralph Weissleder[1,3,4,5], Jung Hyun Jo[2*], Seungmin Bang[2*], Hyungsoon Im[1,4*]

[1] Center for Systems Biology, Massachusetts General Hospital, Boston, MA, USA
[2] Division of Gastroenterology, Department of Internal Medicine, Severance Hospital, Yonsei University College of Medicine, Seoul, Republic of Korea
[3] Cancer Center, Massachusetts General Hospital, Harvard Medical School, Boston, MA, USA.
[4] Department of Radiology, Massachusetts General Hospital, Boston, MA, USA
[5] Department of Systems Biology, Harvard Medical School, Boston, MA, USA

†These authors contributed equally to the manuscript.

*Corresponding authors:
Hyungsoon Im (im.hyungsoon@mgh.harvard.edu)
Seungmin Bang (BANG7028@yuhs.ac)
Jung Hyun Jo (JUNGHYUNJO83@yuhs.ac)

Supplementary Methods:

Experimental setup

The FLEX chip characterization (Figure 2) was performed using an upright microscope (Zeiss, Axio Imager 2) which is equipped with a 40x NA 0.95 objective lens (Plan-Apochromat Corr D=0.25 M27) and a multicolor LED light source (Zeiss, Colibri 7 FR-R[G/Y]BV-UV). The images of the rest experiments were acquired using an inverted microscope (Nikon, Eclipse Ti), a 40x NA 0.95 objective lens (Nikon, MRD70470), and a mercury vapor lamp-based light source (Excelitas technologies, X-Cite 120Q). The equipped fluorescence filter information is summarized in Table S2.

Spectrum measurement

The reflection spectrum was measured by a Nikon 50i upright microscope using a 10x (N.A.= 0.1) objective lens. A halogen light source was illuminated by Kohler illumination and reflected light was collected at a camera port using a convex lens (f = 25mm, Thorlabs). A multimode fiber was located at the focal point of the lens, and the signal was acquired by a USB spectrometer (Ocean Optics).

Chip characterization

1 wt % polyvinyl alcohol (PVA, MW. 13 000-23 000; Sigma-Aldrich) was prepared in water and mixed using a microwave oven. AlexaFluor 488, 555, and 647 dyes were diluted to 50 μM. The dye-containing PVA films were deposited by spin-coating at 3000 rpm, and the thickness is estimated at ~25 nm.

Sample preparation for proteomics

Proteomic analysis, including LC-MS/MS, was performed at the Korea Basic Science Institute (Rep. of Korea, Chungbuk). Each sample was digested in an S-Trap mini spin column (Protifi, USA) according to the manufacturer's instructions. Extracted EVs were homogenized for 20 sec by 5% SDS in 50 mM TEAB. Briefly, 100 μg of proteins was heated to 95°C for 5 min, reduced with 5 mM TCEP (final concentration) for 1 hr at 60°C, and alkylated with iodoacetamide at a final concentration of 20 mM in the dark for 40 min. The alkylated proteins were acidified by adding phosphoric acid to a final concentration of 1.2% and mixed with six volumes of binding buffer (90% methanol; 100 mM TEAB; pH 7.1). After gentle mixing, the protein solution was loaded onto the filter and centrifuged at 4,000 × g for 30 sec. Then the samples were washed two to three times with a 90:10 methanol: TEAB (50 mM) solution and digested with trypsin gold (Promega) at 37°C for overnight at a protein-to-enzyme ratio of 10:1 (w/w). Peptides were eluted stepwise with three elution buffers at a volume of 200 μL each with one more repeat, including 50 mM TEAB in water, 0.2% formic acid in water, and 50% acetonitrile/0.2% formic acid in water.

Tandem mass tag (TMT) labeling and high pH RPLC fractionation

Peptide samples (80 μg each) were labeled with TMT reagent (Thermo Scientific, Rockford, IL) according to the manufacturer's instructions. Each TMT channel powder was freshly dissolved in anhydrous acetonitrile (ACN) with a ratio of 0.8:41 (w:v, mg:μL). After incubation for 1 hr at room temperature, the reaction was quenched by adding 8 μL of 5% hydroxylamine, and the

labeled peptide samples were incubated for 15 min. 2.5% of TMT-labeled peptides from each channel were prepared for LC-MS/MS analysis for determining labeling efficiency before pooling. Pooled TMT-labeled peptides from 6 channels were desiccated by Speed-Vac for High-pH fractionation. A high pH reversed-phase peptide fractionation kit (Thermo Fisher Scientific) was used to fractionate TMT-labeled peptides by increasing acetonitrile step-gradient elution. First, the column was equilibrated with acetonitrile and 0.1% trifluoroacetic acid (TFA). Second, the mixed labeled peptide samples and pure water were loaded and desalted by low-speed centrifugation. Finally, the column was combined with high-pH acetonitrile solution with increasing concentration. The peptides were subjected to gradient elution, and each eluted peptide sample was vacuum dried.

Supplementary Figures:

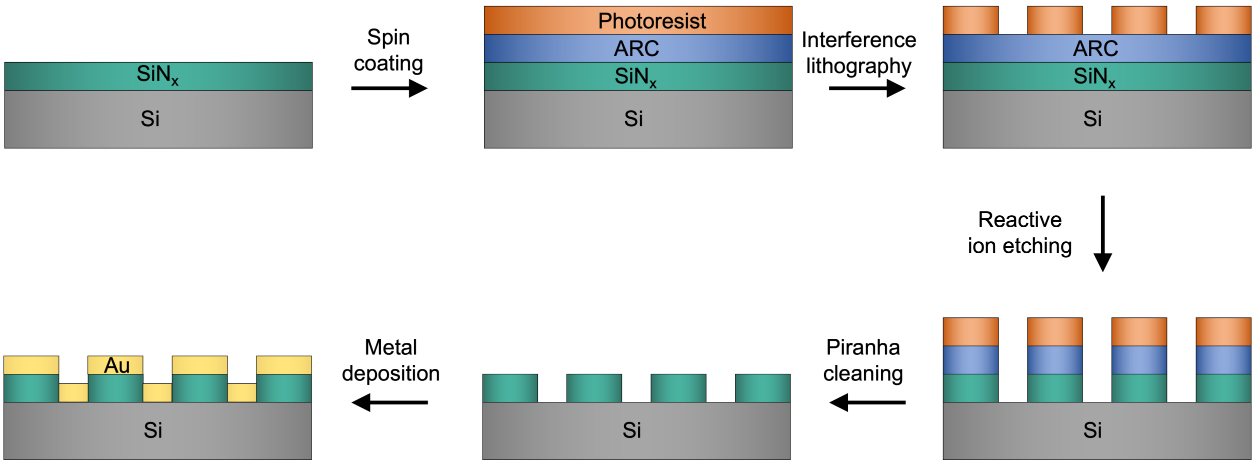

Figure S1. Chip fabrication schematic. Starting material is a 4-inch Si wafer with a 200-nm thick, low-stress silicon nitride (SiN_x) layer deposited by low-pressure chemical vapor deposition (LPCVD). After anti-reflection coating (ARC) and spin-coating of a negative photoresist, two orthogonal grating images were exposed to the photoresist and made periodic nanowell patterns. Subsequent reactive ion etching with CF_4 transferred the hole patterns into the SiN_x layer. The remaining resists were removed by piranha cleaning. Depositing 100-nm thick Au with a 5-nm Ti adhesion layer produced periodic Au nanowell arrays.

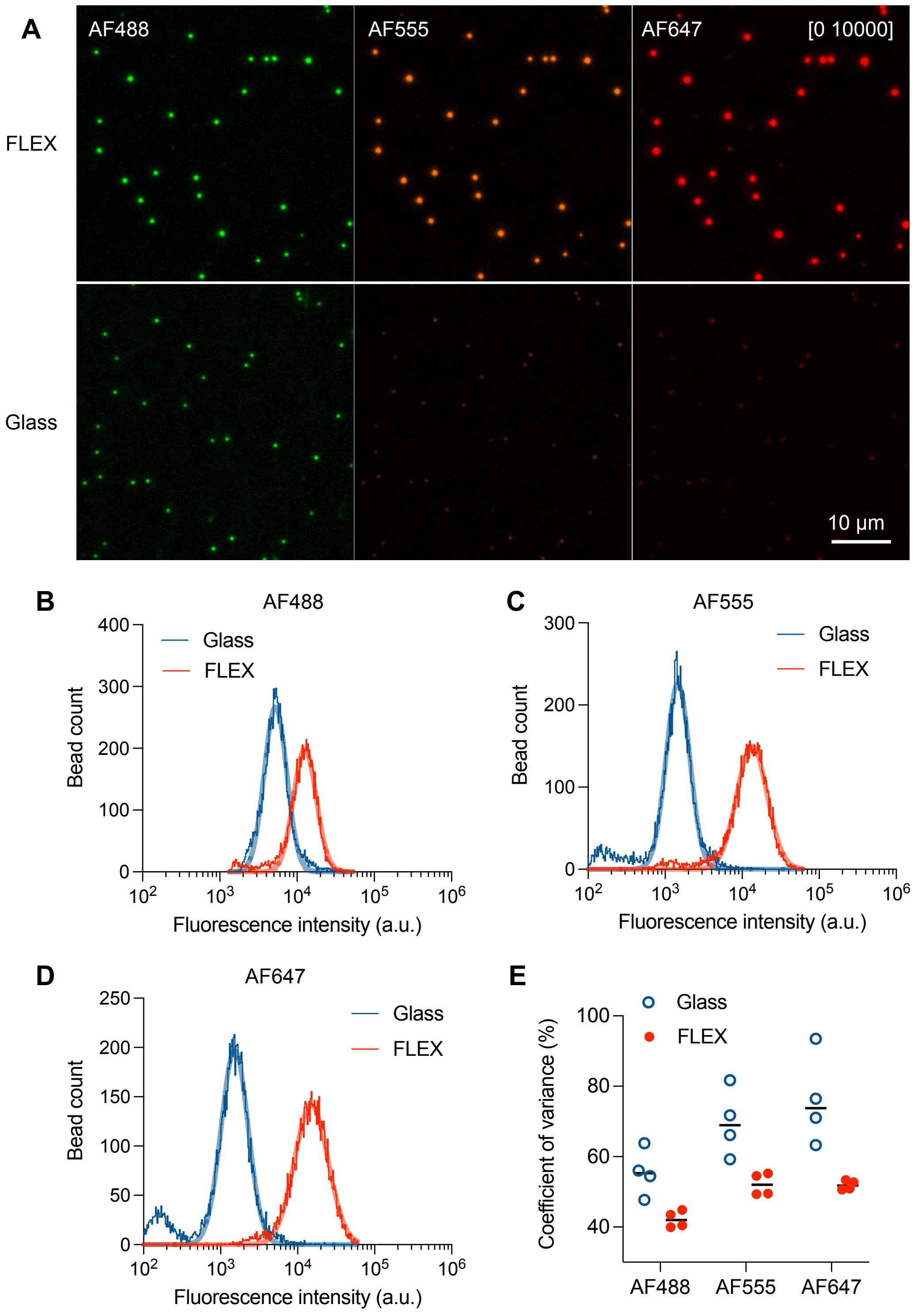

Figure S2. Multi-channel fluorescence imaging of 100 nm multi-fluorescence beads on glass and FLEX substrates (Invitrogen, TetraSpeck). A. Fluorescence images of beads placed on glass and FLEX substrates in AF488, AF555, and AF647 channels. **B-D.** Histogram analysis of fluorescence intensities in (B) AF488, (C) AF555, and (D) AF647. **E.** Coefficients of variance of bead intensities on glass and FLEX substrates.

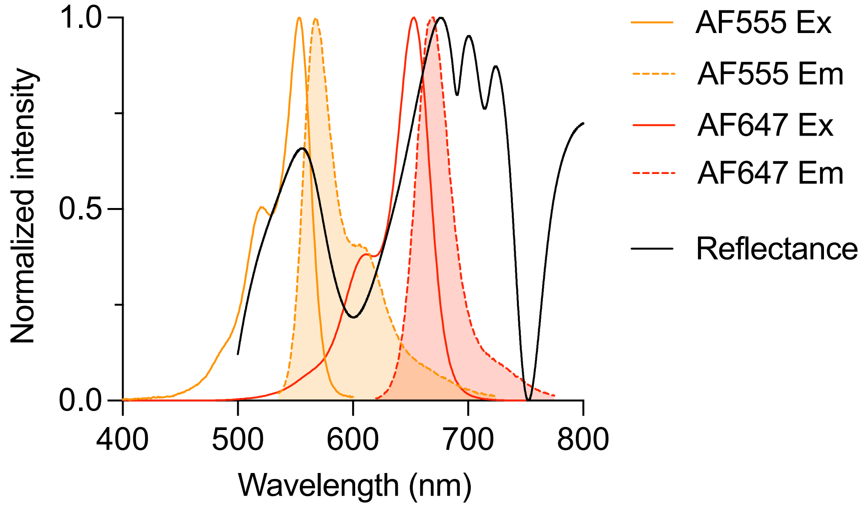

Figure S3. The reflectance spectrum of the periodic nanowell array (200 nm in diameter and 500 nm periodicity) overlapped with the excitation and emission spectra of AF555 and AF647 fluorophores.

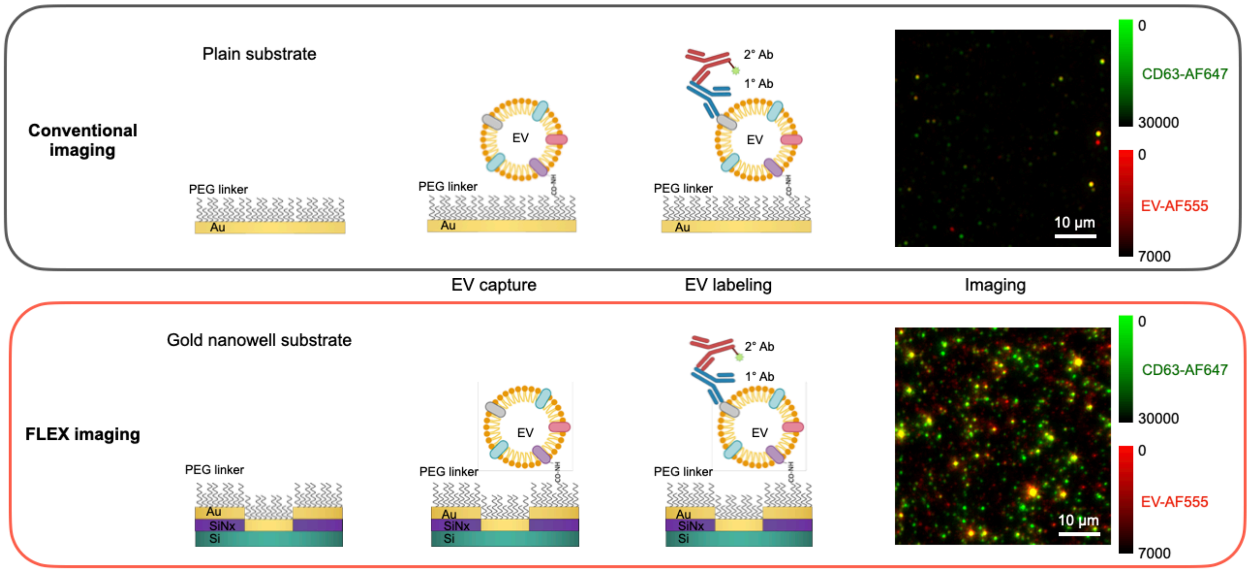

Figure S4. Comparison between conventional EV fluorescence and FLEX imaging. The overall procedures (substrate preparation, EV capture, labeling) are the same, but the only difference is using a gold nanowell substrate instead of plain gold or glass substrates. The gold nanowell substrate, however, significantly amplifies EVs' fluorescence signals, detecting a larger number of EVs than plain substrates.

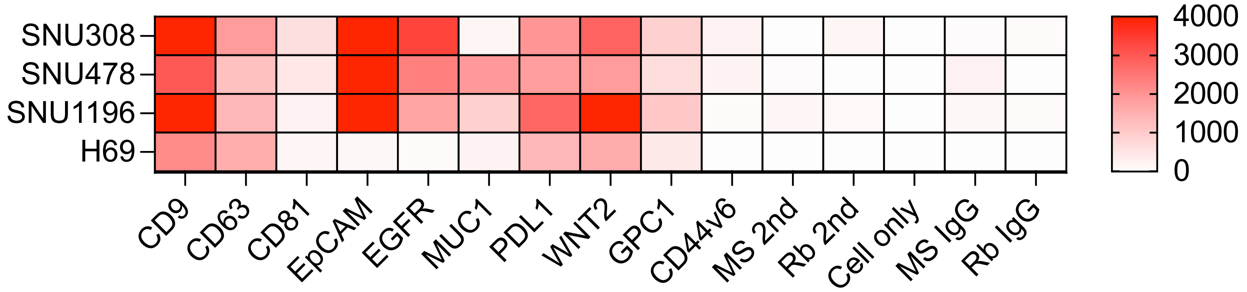

Figure S5. Cell flow cytometry data. Representative heatmap showing the marker expressions of flow cytometry analysis from normal or CCA cell line. Read 10,000 cells and display the number of positive cells for individual antibodies.

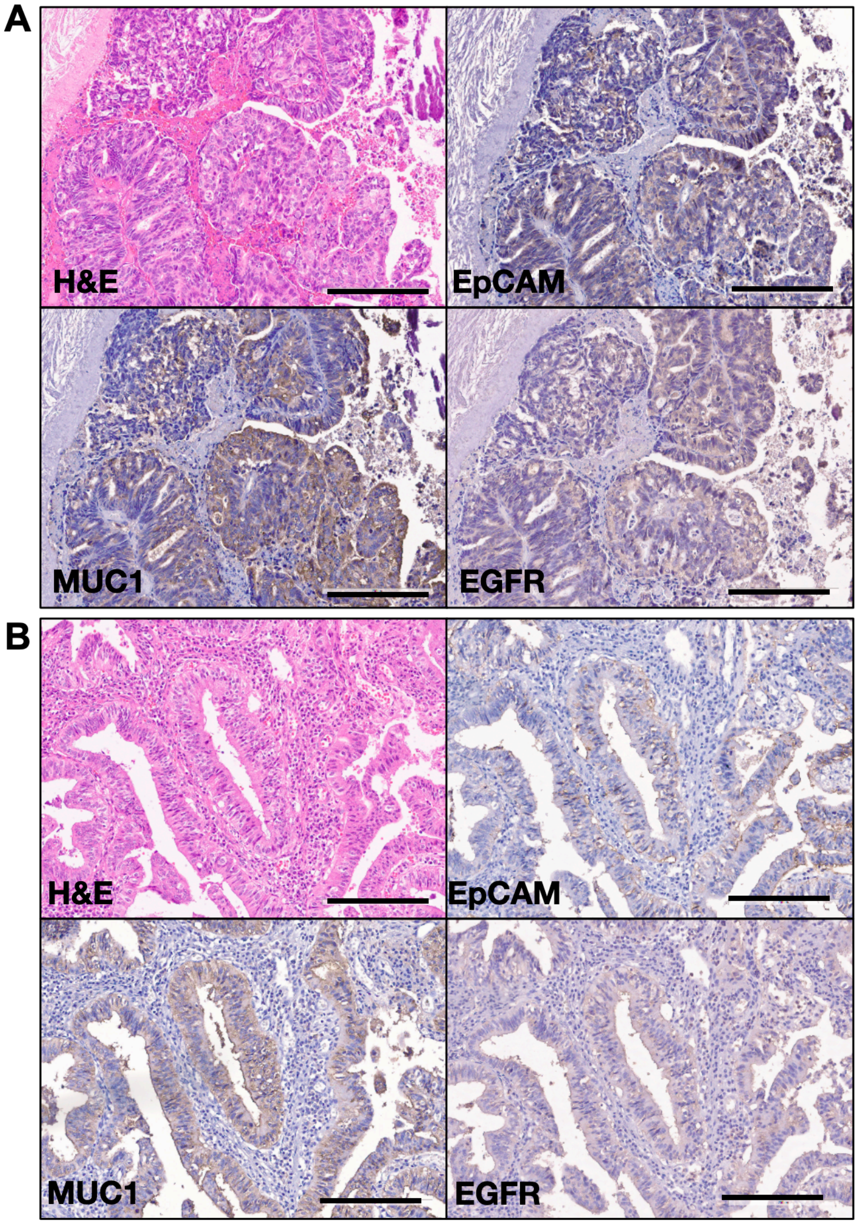

Figure S6. Patient tissue IHC. Hematoxylin-eosin (H&E) and EpCAM, MUC1, and EGFR immunohistochemical staining of two patients (A, B)show high expression of the three markers in the tissue of cholangiocarcinoma patients. Scale bars = 200 μm.

A

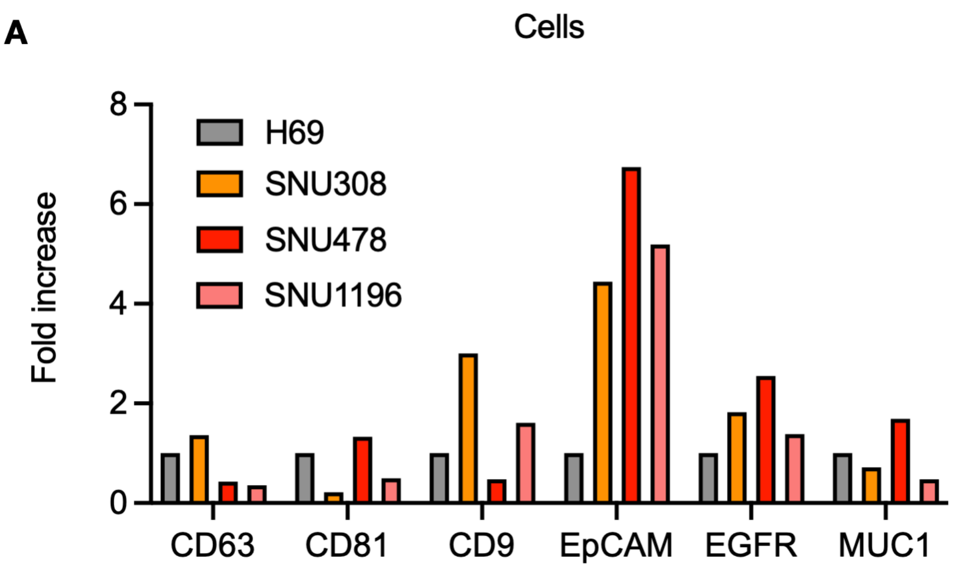

B

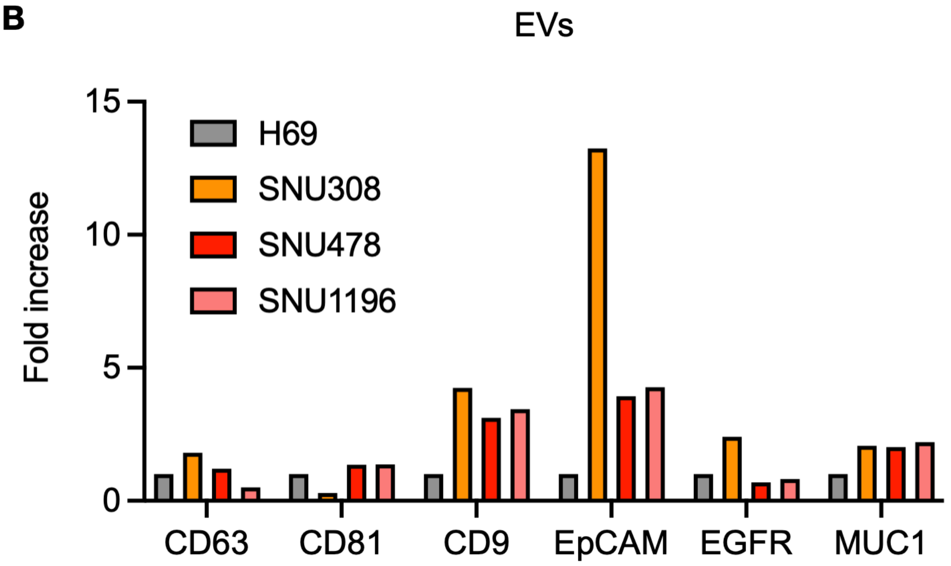

Figure S7. Proteomic analysis on cells and EVs. The LC/MS analysis shows elevated levels of EpCAM, EGFR, and MUC1 in cholangiocarcinoma cell lines (SNU308, SNU478, SNU1196, A) and their EVs (B). The data were normalized and shown as fold change over the marker level of the normal bile duct cell line (H69) and their EVs, respectively.

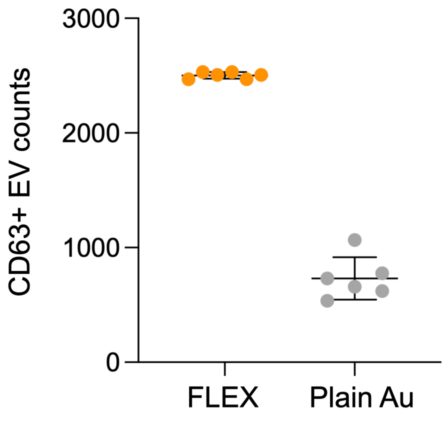

Figure S8. Numbers of CD63-positive EV counts detected with FLEX and plain Au substrates.

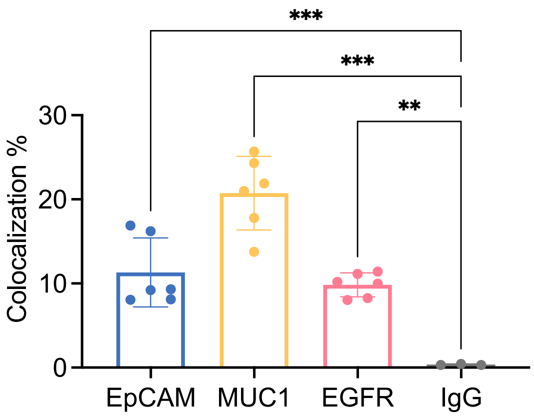

Figure S9. Colocalization percentile between EV and marker channels. EVs from the SNU308 cell line show significantly higher positive rates for EpCAM, MUC1, and EGFR than IgG isotype control. P values were calculated by Mann-Whitney unpaired *t*-test (**$P < 0.01$ and ***$P < 0.001$).

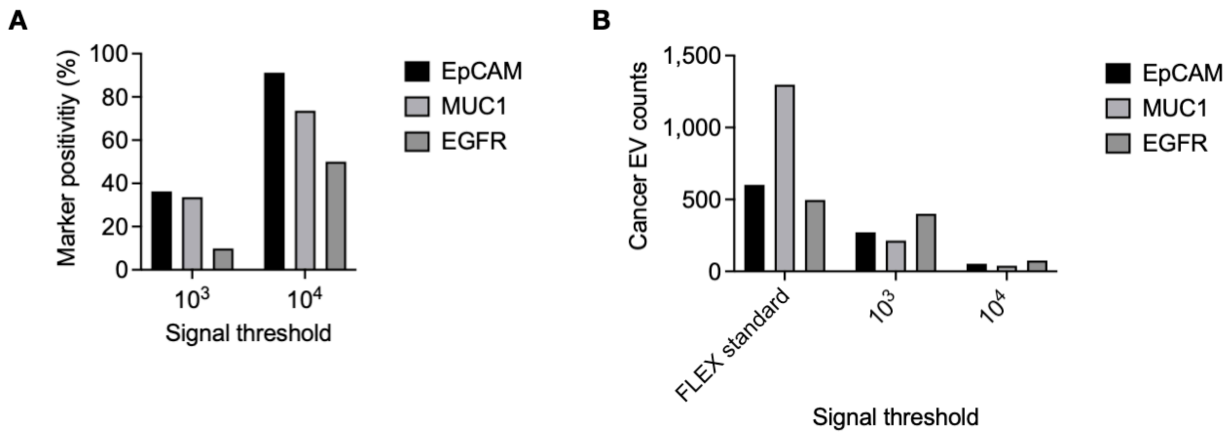

Figure S10. Marker positive rate with different threshold values. A. The marker positivity rates increase with a higher signal threshold value (i.e., only considering EVs with high AF555 signals). **B.** Detected tEV counts with the standard threshold in the FLEX assay (defined by mean + 3 times standard deviation of IgG signal) and higher thresholds (10^3 and 10^4).

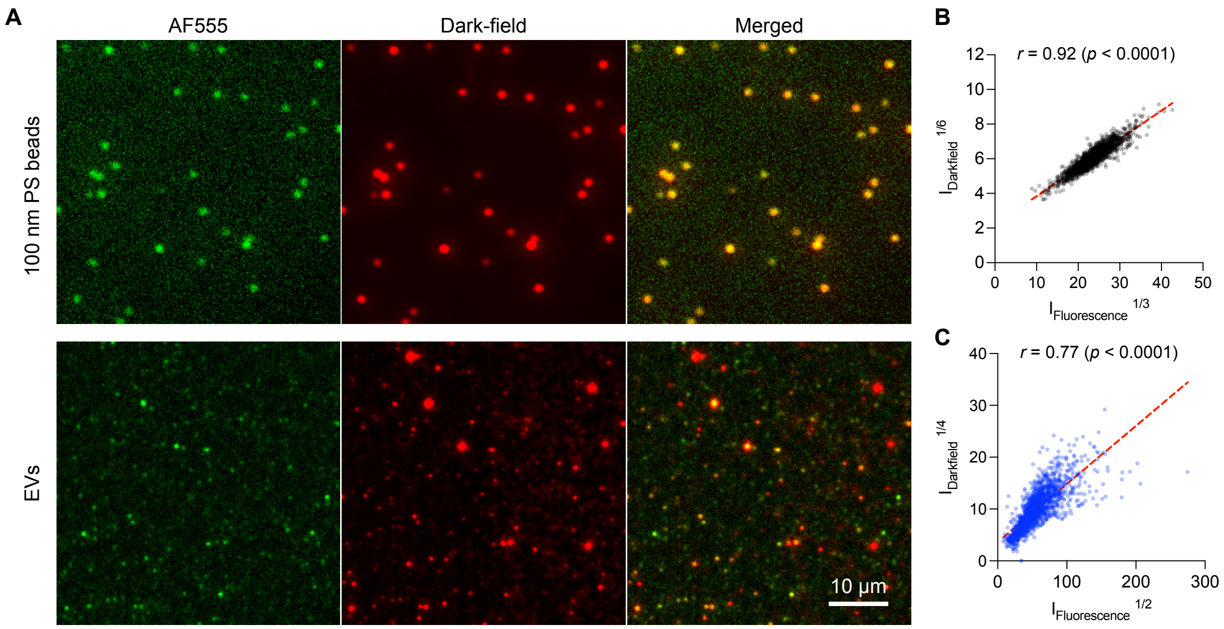

Figure S11. Correlation between dark-field scattering and fluorescence signals. A. Fluorescence and dark-field scattering images of 100 nm polystyrene (PS) beads and EVs labeled by TFP-AF555. **B-C.** Correlation between dark-field and fluorescence intensities for PS beads (B) and EVs (C). The Pearson correlation coefficients are shown on the plot with p-values. The red dot lines show linearly fitted curves.

In testing and optimizing the TFP labeling, we established a correlation between TFP signals and dark-field light scatterings of EVs. The dark-field scattering was measured using the CytoViva darkfield illumination system and a 40x NA 0.9 objective. As shown in Figure S11, TFP-AF555 signals are aligned well with dark-field light scattering for both polystyrene (PS) beads and EVs. We used different metrics for their correlation because of the structural difference between PS beads and EVs. PS beads have uniform materials and dye distribution in the sphere, while EVs are considered hollow shell structures (Vogel et al., J. Extracell Vesicles, 2021;10:e12052). Therefore, EVs' scattering intensities follow Rayleigh-Gans approximation ($I \propto d^4$), while beads' scattering intensity scales with d^6. For fluorescence intensity, TFP labeling applies to the surfaces for EVs (d^2), while fluorescence beads contain the dyes uniformly in the spheres (d^3). Thus, we made scattering plots of $I_{dark}^{1/6}$ vs. $I_{Fluorescence}^{1/3}$ for PS beads and $I_{dark}^{1/4}$ vs. $I_{Fluorescence}^{1/2}$ for EVs. The Pearson correlation coefficients (r = 0.92 for beads and r = 0.77 for EVs) show good correlations between fluorescence and dark-field intensities that are proportional to the size of particles.

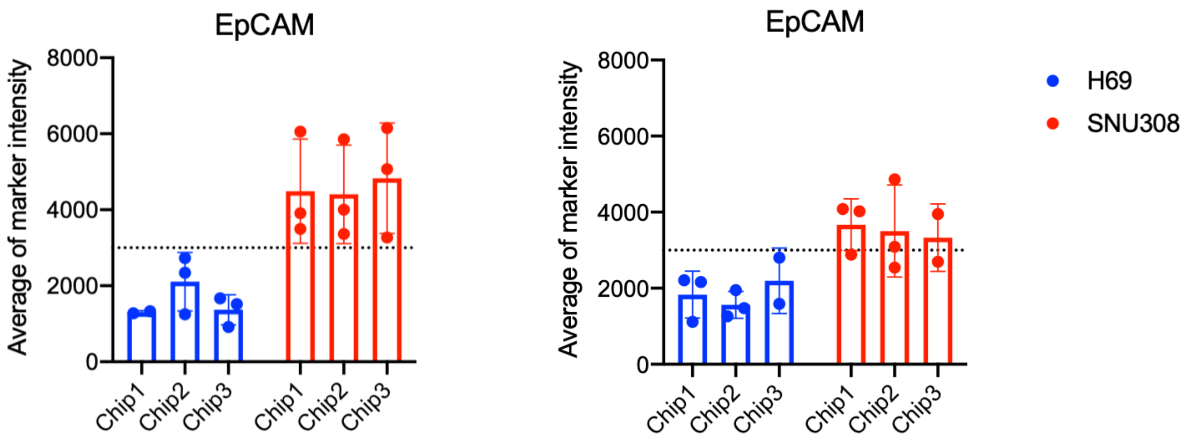

Figure S12. Repeatability and reproducibility tests for the FLEX assay. Three duplication measurements were performed on three different chips for EpCAM detection on EVs from SNU308 and H69 cell lines. Two independent measurements were performed for a reproducibility test. The coefficient of variation (CV) from three different chips was 4.8% for positive controls, while negative controls were below threshold values. The specificity for EpCAM measurements was reproduced in the two independent tests. Each experiment was replicated 3 (n=3).

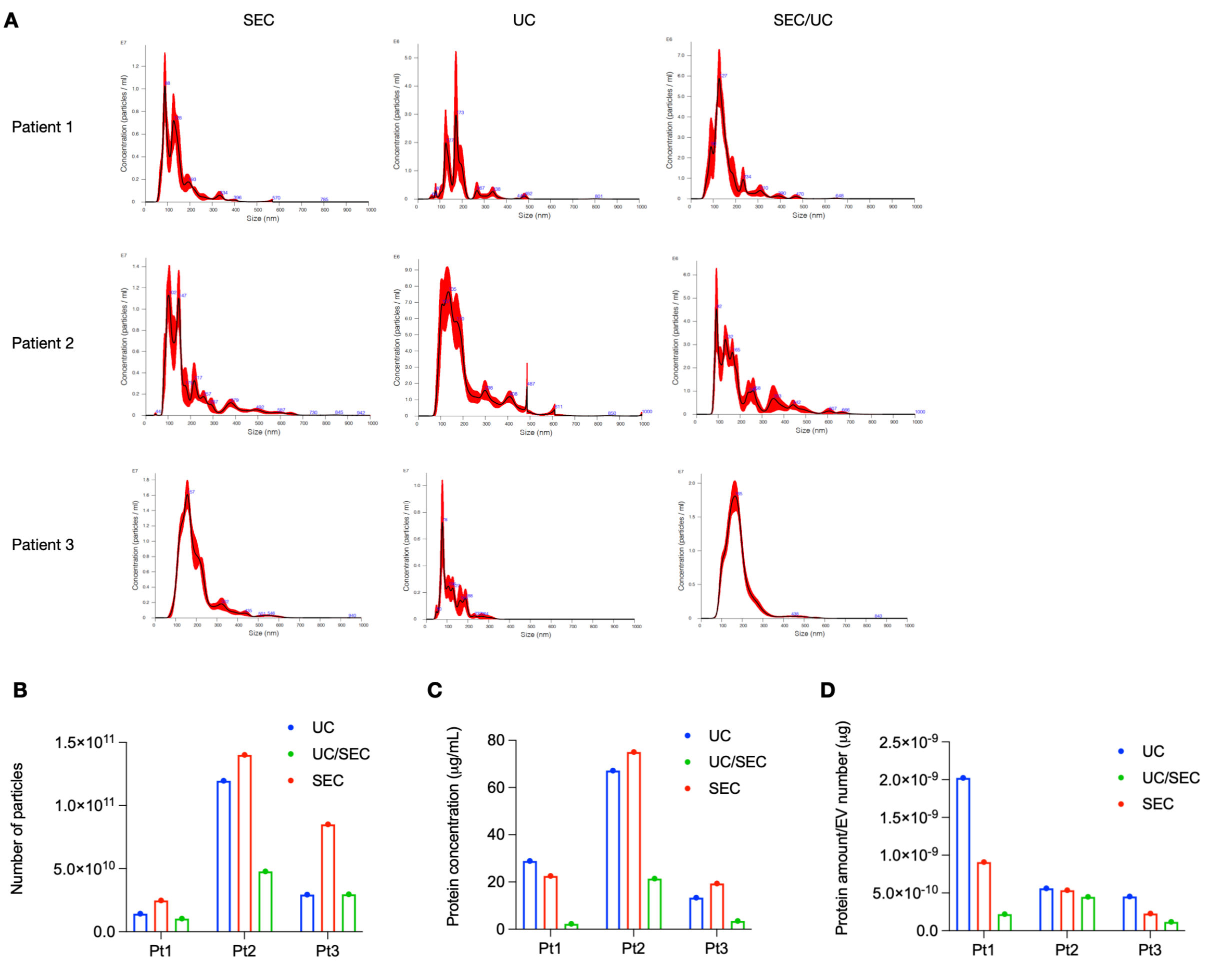

Figure S13. Comparison of three bile-derived EV isolation methods. A. Nanoparticle tracking analysis of bile-derived EVs from three patients using size-exclusion chromatography (SEC), ultracentrifugation (UC), and their combination (SEC/UC). **B-D.** The numbers of total particles (B), protein concentrations (C), and protein amount (µg) per EV for bile-derived EV samples were isolated by the three methods.

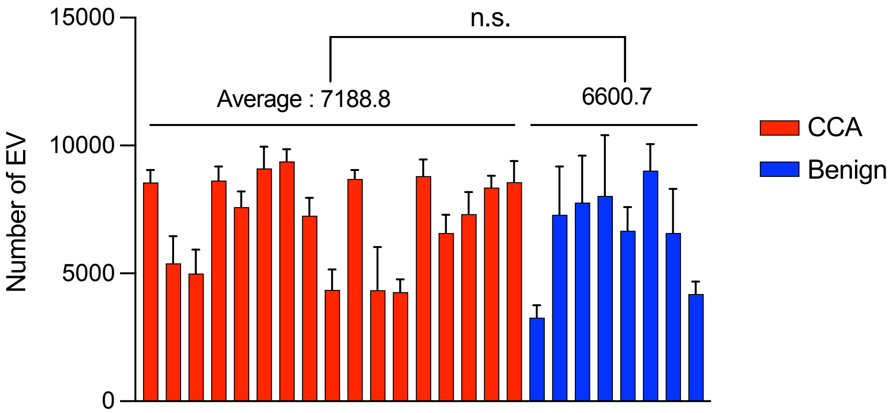

Figure S14. Detected EV counts in bile samples. The error bar shows the standard deviation from 6 repeated measurements. The difference between groups was not statistically significant (p = 0.34, Mann-Whitney test).

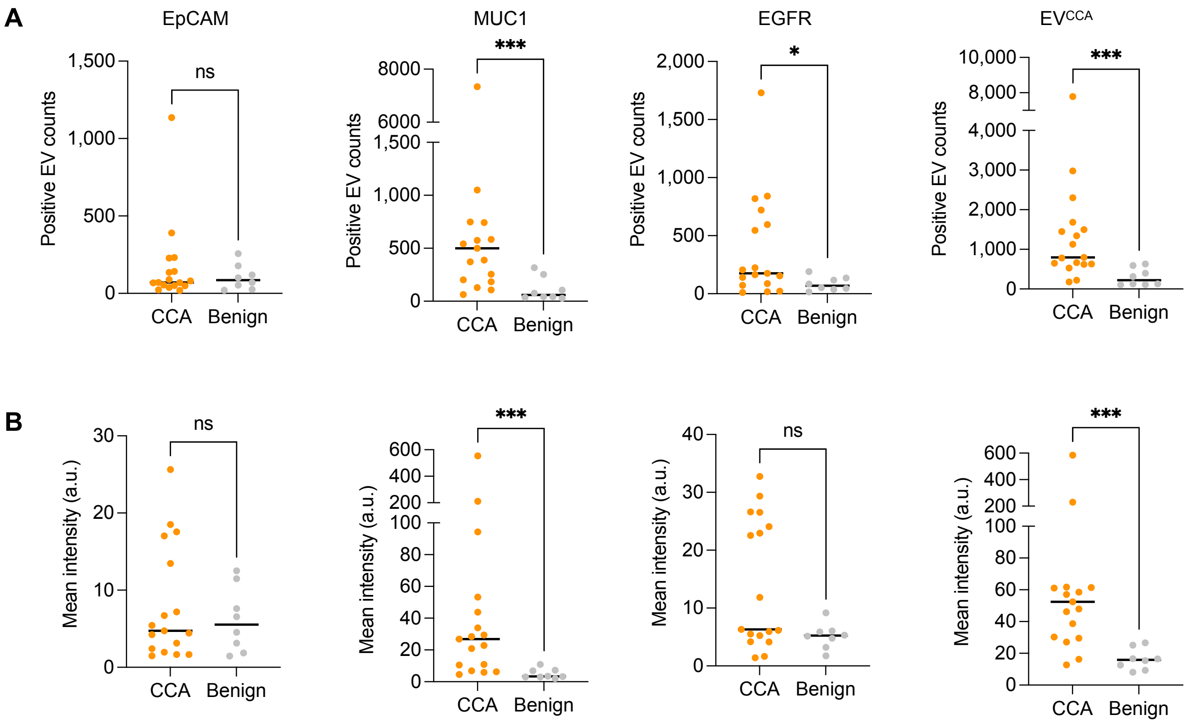

C

	EpCAM	MUC1	EGFR	EV^{CCA}
Marker-positive EV count	0.511	0.904	0.757	0.912
Mean intesnsity	0.537	0.897	0.713	0.934
FLEX signal	0.566	0.890	0.721	0.927

Figure S15. Comparison of marker-positive EV counts and intensities between cholangiocarcinoma (CCA) and benign patient groups. A. Marker-positive EV counts. **B.** Mean intensities of marker-positive EVs. **C.** Area under the curve (AUC) values for marker-positive EV count, mean intensity, and FLEX signal (i.e., total intensities of marker-positive EVs). P values were calculated by Mann-Whitney unpaired *t*-test (***$P < 0.001$).

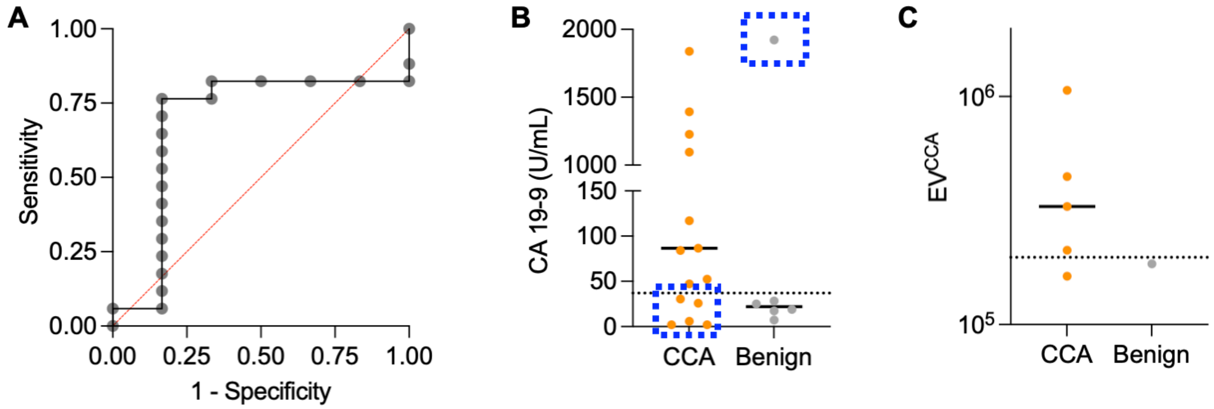

Figure S16. A. Receiver operating characteristic (ROC) curve for CA19-9. **B.** CA19-9 levels between CCA and benign groups. **C.** FLEX analysis using EV^{CCA} signature for six patients misdiagnosed with CA19-9 highlighted as blue dashed boxes in (B). 5 out of 6 patients were correctly diagnosed with the FLEX assay.

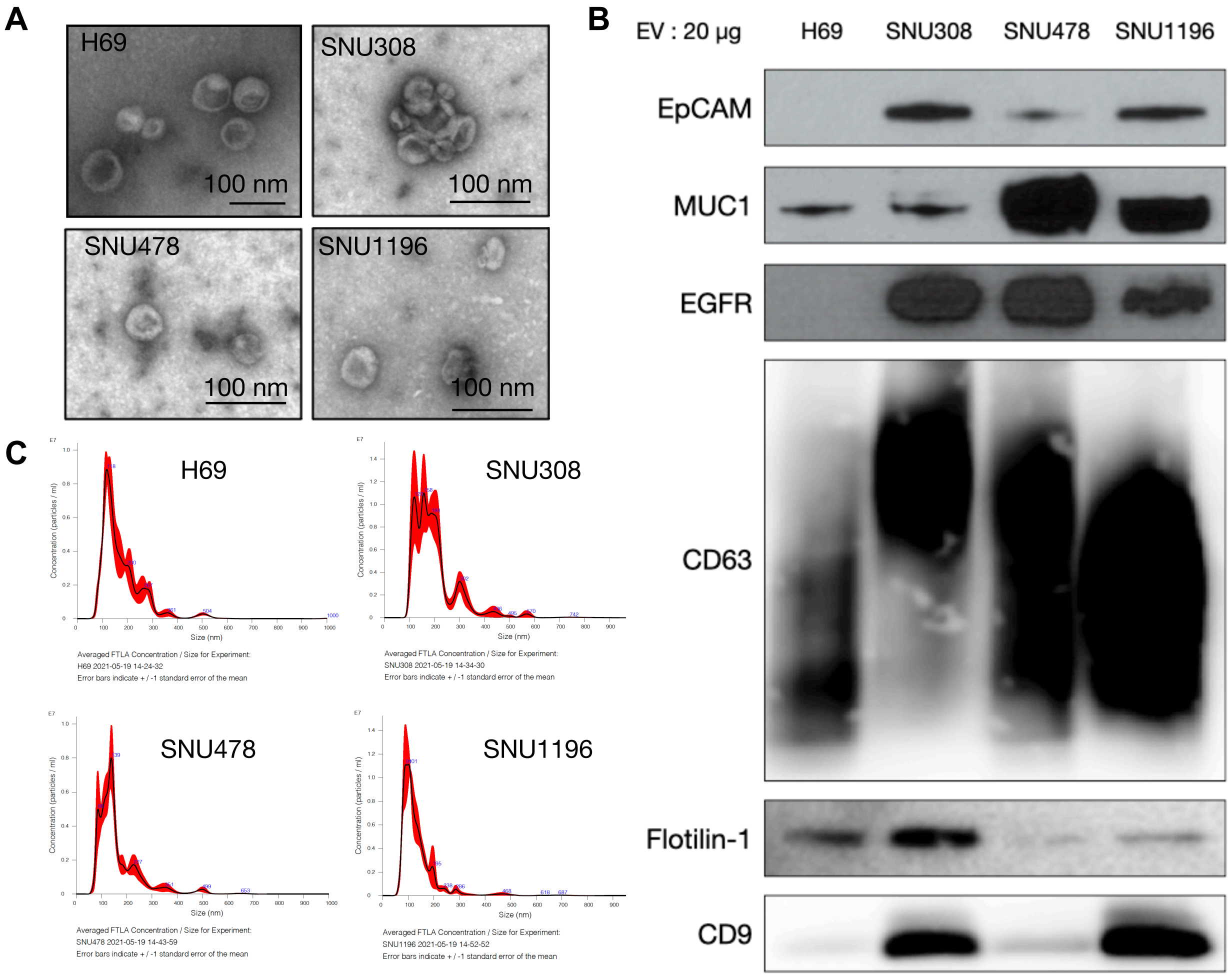

D

H69	SNU308	SNU478	SNU1196	PBS
4.31E+11	7.45E+11	3.54E+11	4.43E+11	N.D. (<1.91E+06)

Figure S17. EV characterization derived from BTC cell lines. A. Transmission electron micrograph of isolated EVs. **B.** Expressions of EV marker (CD63, Flotilin-1, CD9) and EV tumor markers (EpCAM, MUC1, EGFR) measured with Western blot with EV lysis. **C.** EV concentrations and size distributions measured with nanoparticle tracking analysis (NTA). **D.** The average EV concentrations were summarized for cell line-derived EVs (*n* = 4). The presence of background particles in the PBS buffer solution was also investigated. From 5 repeated measurements, three measurements showed no particle presence, and two measurements showed only 0.2 particles per frame. The calculated average concentration was below the limit of detection of particles of NTA (1.7 × 10^7 particles/mL, NanoSight LM-10, Steppert et al., J Chromatogr A. 2017;1487:89-99). We, therefore, concluded EVs are non-detectable (N.D.) in the PBS solution.

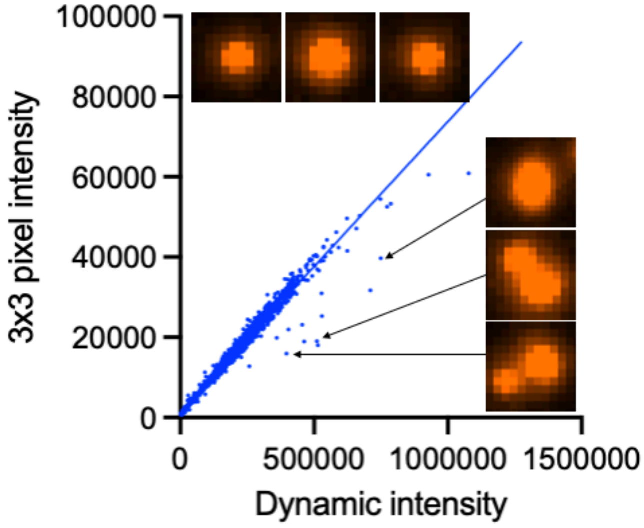

Figure S18. Comparison of fluorescence intensity values of 100-nm polystyrene beads using the 3x3 pixel intensity and dynamic window methods.

Table S1. Antibodies used in this study

Antibody (FLEX/EV flow cytometry)	Vendor	Cat No.
**Primary antibodies**		
CD63 antibody (monoclonal)	Ancell	215-820
EpCAM antibody (monoclonal)	Invitrogen	MA5-12436
MUC1 antibody (monoclonal)	Invitrogen	MA1-06503
EGFR antibody (monoclonal)	Invitrogen	MA5-13070
**Isotype**		
Isotype control mo IgG1,k	Invitrogen	14-4714-82
**Secondary Antibody**		
Goat anti-mouse IgG 2nd Antibody, Alexa 647	Invitrogen	A-21235
**Antibody (Tissue and cellular anlaysis)**	Vendor	Cat No.
**Primary antibodies**		
EpCAM antibody (monoclonal)	abcam	ab20160
MUC1 antibody (monoclonal)	Fitzgerald	10-M93B
EGFR antibody (monoclonal)	Santa Cruz	sc365829
**Secondary Antibody**		
Goat anti-rabbit/mouse IgG 2nd Antibody	Dako	K5007

Table S2. Fluorescence filter information

Microscope	Fluorescence channel	Excitation (nm)	Dichroic mirror (nm)	Emission (nm)
Zeiss	AF488	480/30	505	535/40
Zeiss	AF555	540/25	565	605/55
Zeiss	AF647	620/50	655	690/50
Zeiss	Cy7	710/75	760	810/90
Nikon	AF488	480/30	505	535/45
Nikon	AF555	540/25	565	605/55
Nikon	AF647	620/60	660	700/75
